# On the definition of a self-sustaining chemical reaction system and its role in heredity

**DOI:** 10.1186/s13062-020-00269-0

**Published:** 2020-10-06

**Authors:** Yu Liu

**Affiliations:** Institut Mittag-Leffler, Auravägen 17, Djursholm, 18260 Sweden

**Keywords:** Definition of life, Origin of life, Autocatalysis, Self-replication, Non-equilibrium, CSTR, Molecular trigger, Molecular seed, Limited heredity

## Abstract

**Background:**

The ability to self-sustain is one of the essential properties of life. However, a consistent and satisfying definition of self-sustainability is still missing. Currently, self-sustainability refers to either “no-intervention by a higher entity” or “regeneration of all the system’s components”. How to connect self-sustainability with heredity, another essential of life, is another problem, as they are often considered to be independent of each other. Last but not least, current definitions of self-sustainability failed to provide a practical method to empirically discern whether a chemical system is self-sustaining or not.

**Results:**

Here I propose a definition of self-sustainability. It takes into account the chemical reaction network itself and the external environment which is simplified as a continuous-flow stirred tank reactor. One distinct property of self-sustaining systems is that the system can only proceed if molecular triggers (or called, seeds) are present initially. The molecular triggers are able to establish the whole system, indicating that they carry the preliminary heredity of the system. Consequently, life and a large group of fires (and other dissipative systems) can be distinguished. Besides, the general properties and various real-life examples of self-sustaining systems discussed here together indicate that self-sustaining systems are not uncommon.

**Conclusions:**

The definition I proposed here naturally connects self-sustainability with heredity. As this definition involves the continuous-flow stirred tank reactor, it gives a simple way to empirically test whether a system is self-sustaining or not. Moreover, the general properties and various real-life examples of self-sustaining systems discussed here provide practical guidance on how to construct and detect such systems in real biology and chemistry.

**Reviewers:**

This article was reviewed by Wentao Ma and David Baum.

## Background

NASA defines life as a self-sustaining chemical system capable of undergoing Darwinian evolution [1, 2]. Here, “self-sustaining” implies that a living system should not need continuous intervention by a higher entity (e.g. a graduate student or a god) to continue as life [1]. Another popular definition of life comes from the concept of *autopoiesis*: a system is said to be living if it is capable of self-sustaining owing to an inner network of reactions that regenerate all the system’s components [3]. But the phrase “self-sustaining” refers to “no-intervention” in the former definition, but “regeneration” in the latter.

In the fields that relate to essentials of life, such as biochemistry [4, 5], molecular biology [6], network autocatalysis [7–11], and non-equilibrium thermodynamics [12, 13], this phrase “self-sustaining” or “self-sustainability” is being frequently used in a vague and ambiguous manner, but mostly refers to the two different aspects mentioned above, although they are not necessarily contradictory.

In the “no-intervention” school, for example, a designed RNA enzyme system that underwent exponential amplification was said to be self-sustaining in the sense that the amplification could be continued indefinitely [4]. A chemical reaction loop that was invented to convert amines to alcohols was said to be self-sustaining in the sense that products were created, purified, and isolated without manual operations [5].

On the other hand, the “regeneration” school focuses more on the mechanism that leads to self-sustainability [8–11, 14–22]. For example, Piedrafita et al. referred “self-sustainability” to “metabolic closure” that all of the catalysts essential for the survival of an organism have to be produced internally [14, 15, 17]. In the *reflexively autocatalytic and food-generated* (RAF) theory, “self-sustaining” was referred to that each molecule in a chemical network can be produced starting from the food source [8, 18, 19, 23]. To be noticed, the *chemical organisation theory* proposed a rigorous definition of “self-sustaining” (self-maintaining, in their words) [10, 20, 21]: A set of molecules is called semi-self-maintaining if topologically all molecules that are consumed are also produced; it is further called self-maintaining if the stoichiometry of the network makes the production rate of each molecule strictly nonnegative.

Although the definition in the chemical organisation theory is rigorous, it has shortcomings. Firstly, it is merely a topological description. Although the topology of the coupled network is important, the strength of the couplings (namely the reaction rates) could completely change the behaviours of the whole system [14, 24–26]. Secondly, it is defined with respect to a set of molecules, rather than a system. For a reaction system that involves *N* molecule types, it can be partitioned into $ {N \choose 0} + {N \choose 1} + \dots + {N \choose N} = 2^{N}$ sets of molecules, each of which may or may not be self-maintaining, based on their definition. However, different sets of molecules cannot be physically isolated as they are all involved in one system. Thirdly, this definition is too stringent: It requires all molecules that are consumed to be also produced. However, even for a living system which should be categorised as a self-sustaining system, it cannot produce the resource molecules it needs.

Another crucial point about self-sustainability is how to connect it with heredity, another essential of life. Currently, they are often considered to be independent of each other. So origins of life require one origin of self-sustainability and an independent origin of heredity, respectively. That is also why the theory stating that life began with a self-sustaining chain of chemical reactions, without the requirement for genetic information, has been heavily questioned [27–30]. But what if self-sustainability naturally guarantees heredity, or at least preliminary heredity (as we shall discuss at the end of this paper)?

The last point is how to empirically discern whether a chemical system is self-sustaining or not, which is not a trivial question at all. The definitions mentioned above are all based on the complete topological information of a chemical reaction system, including all the reactants, products, intermediates and how they are connected via reactions. However, in most real chemical experiments, only partial information is known, or even, all we know are what has been put into the system and what has been produced. The complete information is almost impossible. To get around this problem, I will base the definition of self-sustainability only on the information of what has been put into and produced from the system.

Furthermore, to connect real experiments, I will define self-sustainability in the context of a continuous-flow stirred tank reactor (CSTR) particularly, which is commonly used in chemical engineering [31–33]. Nevertheless, this definition will not lose its generality, since whether a system has the ability to self-sustain is an intrinsic property of the system itself, which we shall see later.

This paper is organised as follows. Firstly, the theoretical setup is introduced via a detailed example, followed by the formal definition of self-sustainability. And then, the general properties of a chemical system that has the potential to be self-sustaining are discussed, to give guidance for constructing or finding such systems in real biology and chemistry. After that, various self-sustaining systems that are observed in labs and real living systems are shown. In the “Discussion” section, besides some comments on the definition, two more questions are discussed: why self-sustaining systems have preliminary heredity, and why life and fire are distinct in terms of self-sustainability. The conclusions are drawn in the end.

## Theoretical setup

We first introduce one terminology: A *chemical reaction network* (CRN) comprises a set of reactants, a set of products, and how they are linked via reactions [34]. To investigate CRNs as broadly as possible, we employ the artificial chemistry framework that has been developed in a previous paper [35]. All of the CRNs that are constructed based on this framework satisfy basic physical principles such as mass conservation and thermodynamics, and some of them also correspond to real chemical systems. This framework helps us explore CRNs that are unknown to us, but not totally arbitrarily. In the meanwhile, by using the integer notation of molecules instead of complex chemical formulas, the readers are free from lots of chemical details. Nevertheless, the definition of self-sustainability and the discussions in this paper do not suffer from loss of generality.

Here we recap main points of this framework [35]: (1) A molecule is denoted by an integer, $\overline {i}$; (2) Only synthesis reaction and decomposition reaction are possible, and the sums of both sides should be equal, e.g., $\overline {2} + \overline {4} \rightarrow \overline {6}$ and $\overline {8} \rightarrow \overline {1} + \overline {7}$; (3) Each molecule has its own standard Gibbs energy of formation, and each reaction has its own Gibbs energy of activation, which together determine the reaction rate constant of each reaction.

To illustrate how we investigate the dynamics of a CRN, and how we define self-sustainability, we take CRN 1 as an example,

1 $$  \left\{\begin{aligned} \overline{1} + \overline{2} & \rightarrow \overline{3} \\ \overline{1} + \overline{3} & \rightarrow \overline{4} \\ \overline{4} & \rightarrow \overline{2} + \overline{2} \end{aligned}\right.  $$

with the reaction rate constant for each reaction predefined. CRN 1 is a model of the formose reaction which involves the formation of sugars from formaldehyde [35]. Specifically, molecule $\overline {1}$ stands for formaldehyde, $\overline {2}$ for glycolaldehyde, $\overline {3}$ for glyceraldehyde, and $\overline {4}$ for tetrose, respectively.

As mentioned, the chemical system is in a CSTR [31, 32], where the solution is in a tank and gets well-stirred all the time; the solution of the resource molecules continuously flows into the tank, at a fixed flow rate; the solution in the tank is continuously flowed out, at the same rate as the inflow, to keep the volume of the solution fixed. Then, the ordinary differential equations (ODEs) for the mean-field dynamics of CRN 1 can be written as (see Appendix A1 for detailed derivations)

2 $$  \left\{\begin{aligned} \dot{n}_{0} = & ~f_{0} -F' n_{0} / N \\ \dot{n}_{1} = & ~f_{1} -F' n_{1} / N -r_{1} -r_{2} \\ \dot{n}_{2} = & ~f_{2} -F' n_{2} / N -r_{1} +2 r_{3} \\ \dot{n}_{3} = & ~f_{3} -F' n_{3} / N +r_{1} -r_{2} \\ \dot{n}_{4} = & ~f_{4} -F' n_{4} / N +r_{2} -r_{3} \end{aligned}\right.  $$

with

$$\left\{\begin{aligned} r_{1} = & ~\omega_{1} n_{1} n_{2} / (v N) \\ r_{2} = & ~\omega_{2} n_{1} n_{3} / (v N) \\ r_{3} = & ~\omega_{3} n_{4} \\ F' = & ~F -r_{1} -r_{2} +r_{3} \end{aligned}\right. $$

where *n*_*i*_ is the population (*mol*) of molecule $\overline {i}$ in the tank (note that $\overline {0}$ represents the solvent molecule, which never reacts with other molecules); $N = \sum _{i = 0}^{4} n_{i}$ is the total population of molecules; *f*_*i*_ is the constant inflow rate (*mol*/*s*) of molecule $\overline {i}$; $F = \sum _{i=0}^{4} f_{i}$ is the constant overall inflow rate; *r*_*i*_ is the reaction rate (*mol*/*s*) of the *i*th reaction in CRN 1; *ω*_*i*_ is the reaction rate constant (1/*s*) for the *i*th reaction; *F*^′^ is the overall outflow rate (*mol*/*s*); and *v* is a dimensionless constant related to the molar volume of the solution (here we set *v*=0.018), which is explained below.

The derivation of Eq. 2 requires two assumptions (referring to Appendix A1): all molecules are uniformly distributed, and all chemical species have the same molar volume *α* (*L*/*mol*). Consequently, the total volume of the solution is proportional to *N*, and *F*^′^*n*_*i*_/*N* is the outflow rate of molecule $\overline {i}$. In this paper, we set *α* to be the molar volume of water, namely 0.018 *L*/*mol*. This value is where *v* comes from. That means, 1 *mol* of the solution in the tank always takes up 0.018 *L*. These two assumptions make the solution behave like an ideal gas, but just much more condensed. Besides, because of these two assumptions and the CSTR setting, *N* is guaranteed to be the total population initially (thus a constant). So, *n*_*i*_,*r*_*i*_ and *F*^′^ are functions of *t* (which has been omitted to write explicitly); while *N*, *f*_*i*_, *F*, *ω*_*i*_ and *v* are constants.

Finally, for convenience, we denote the molecule population as a vector ***n***=(*n*_0_,*n*_1_,*n*_2_,*n*_3_,*n*_4_), the constant inflow as a vector ***f***=(*f*_0_,*f*_1_,*f*_2_,*f*_3_,*f*_4_), and the outflow as a vector ***f***^***′***^=(*n*_0_,*n*_1_,*n*_2_,*n*_3_,*n*_4_)·*F*^′^/*N*≡***n***·*F*^′^/*N*. We always use Greek letter ***ξ*** to denote initial conditions. We use $\phantom {\dot {i}\!}\boldsymbol {f'}_{\boldsymbol {\xi }, \boldsymbol {f}}$ to denote the outflow under initial condition ***ξ***, given inflow ***f***. We may add a subscript (such as *a*,*b* and *c*) to ***f*** and ***ξ***, to distinguish among different ***f***’s and ***ξ***’s.

**Fig. 1:**
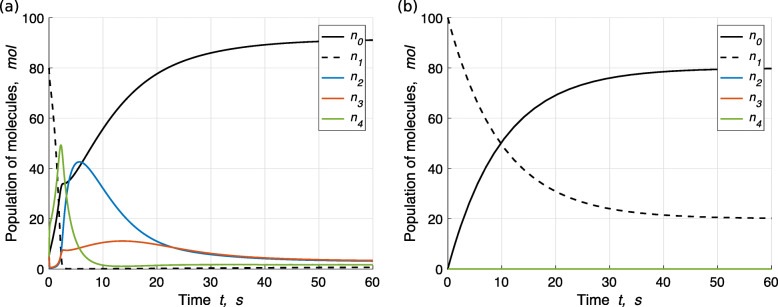
Mean-field dynamics of CRN 1 (formose reaction) in CSTR. The reaction rate constants are *ω*_1_=1,*ω*_2_=0.7 and *ω*_3_=0.4. **a** The initial condition is ***ξ***_*a*_=(*n*_0_,*n*_1_,*n*_2_,*n*_3_,*n*_4_)=(80,5,5,5,5), meaning that there are always *N*=100 *mol* molecules in the solution and thus the total volume of the solution in the tank is *ν*×*N*=1.8 *L*. The inflow is ***f***_*a*_=(*f*_0_,*f*_1_,*f*_2_,*f*_3_,*f*_4_)=(8,2,0,0,0), meaning that *F*=10 *mol* fresh solution flows into the tank per second, 80% (=*f*_0_/*F*) of which is the solvent molecule $\overline {0}$ and 20% of which is molecule $\overline {1}$. It also means that per second, 10% (=*F*/*N*) of the solution in the tank is replaced. After the transient period (*t*>50), the outflow is $\boldsymbol {f^{\prime }}_{\boldsymbol {\xi }_{a}, \boldsymbol {f}_{a}} \doteq (91.5, 0.7, 3.0, 3.2, 1.7)$. **b** The initial condition is ***ξ***_0_=(100,0,0,0,0), and the inflow is ***f***_*a*_, as the same as in (**a**). After the transient period, the outflow is $\boldsymbol {f^{\prime }}_{\boldsymbol {\xi }_0, \boldsymbol {f}_{a}} = (8, 2, 0, 0, 0)$

Given ***f***_*a*_=(8,2,0,0,0), under initial condition ***ξ***_*a*_=(80,5,5,5,5), ODEs 2 can be solved numerically, as shown in Fig. 1a. We see that after a transient period (*t*>50), the system is stationary, i.e., the population of each molecule type does not change. Nevertheless, all of the three reactions in CRN 1 continue to occur for all time, and that is why the outflow is different from the inflow, namely $\phantom {\dot {i}\!}\boldsymbol {f'}_{\boldsymbol {\xi }_{a}, \boldsymbol {f}_{a}} \neq \boldsymbol {f}_{a}$.

Given another initial condition (100,0,0,0,0), the dynamics is shown in Fig. 1b. After the transient period, the outflow and the inflow are identical. The reason is that if molecule $\overline {2}, \overline {3}$ or $\overline {4}$ is not present initially to trigger the system, no reaction can proceed, so no matter what else molecules are in the tank initially they will be washed away, and finally the compositions of the inflow, the outflow and the solution in the tank are identical. We call this type of initial condition that the populations of all other molecules except the solvent molecule are zeros as the zero initial condition, denoted ***ξ***_0_. Now, we can describe the situation shown in Fig. 1b as $\phantom {\dot {i}\!}\boldsymbol {f'}_{\boldsymbol {\xi }_0, \boldsymbol {f}_{a}} = \boldsymbol {f}_{a}$, after the transient period.

## Definition of self-sustainability

Now, we define that

- given inflow ***f***, a CRN (with its the reaction rate constants given) is *self-sustaining* with respect to initial condition ***ξ*** if and only if after the transient period,
  (i) $\phantom {\dot {i}\!}\boldsymbol {f'}_{\boldsymbol {\xi }, \boldsymbol {f}} \neq \boldsymbol {f}$ and
  (ii) $\phantom {\dot {i}\!}\boldsymbol {f'}_{\boldsymbol {\xi }_{0}, \boldsymbol {f}} = \boldsymbol {f}$.

Equivalently, condition (i) says that, given the non-zero initial condition ***ξ***, the outflow converges to a state that deviates from the inflow; while condition (ii) says that, given the zero condition ***ξ***_0_, the outflow converges to a state identical to the inflow.

Condition (i) guarantees that reactions in the tank continue to occur for all time, which makes $\phantom {\dot {i}\!}\boldsymbol {f'}_{\boldsymbol {\xi },\boldsymbol {f}}$ differ from ***f***. Those reactions are able to occur for all time either (a) because the inflow directly results in a sequence of reactions, i.e., the products of reactions in this step constitute all of the reactants of reactions in the next step, so on and so forth, or (b) because all of the reactants needed in this system are regenerated for all time.

Condition (ii) says that for a zero initial condition ***ξ***_0_, the outflow and the inflow are identical. In principle, we need to check this condition for every zero condition. But as the solvent molecule never reacts with other molecules (as defined), its non-zero initial population does not really affect the dynamics of the system, so we just need to check any one of the zero initial conditions. Condition (ii) guarantees that if initially there is no molecule to trigger the system, no reaction can proceed, so that possibility (a) mentioned above is ruled out. The requirement of trigger molecules (or called, seeds) is a crucial property of self-sustaining systems, which will be discussed in details later.

Now we can say that, given the inflow ***f***_*a*_=(8,2,0,0,0), CRN 1 is self-sustaining with respect to the initial condition ***ξ***_*a*_=(80,5,5,5,5). Intuitively and less rigorously, we can interpret a self-sustaining system as such a system that, given an inflow, can constantly regenerate the members of this system if and only if seeded by at least one member of the system.

Note that the concept of self-sustainability only makes sense if we specify the inflow ***f*** and the initial condition ***ξ***, due to the two facts. First, under the same ***ξ***, a CRN may or may not be self-sustaining given different ***f***’s. For example, under ***ξ***_*a*_ as in Fig. 1a, given a different inflow ***f***_*b*_=(8,0,0,2,0), we will have $\phantom {\dot {i}\!}\boldsymbol {f'}_{\boldsymbol {\xi }_{a}, \boldsymbol {f}_{b}} = \boldsymbol {f}_{b}$ because the reactions cannot proceed without $\overline {1}$. So, given ***f***_*b*_, CRN 1 is not self-sustaining with respect to ***ξ***_*a*_. Second, given the same ***f***, only certain types of molecules can trigger a CRN. For example, given ***f***_*a*_ as in Fig. 1a, under a different initial condition ***ξ***_*c*_ where there are 10 of molecule $\overline {5}$ in the tank initially (although in the ODEs, we did not consider molecule $\overline {5}$, but in principle, ***ξ***_*c*_ is a valid initial condition), we will have $\phantom {\dot {i}\!}\boldsymbol {f'}_{\boldsymbol {\xi }_{c}, \boldsymbol {f}_{a}} = \boldsymbol {f}_{a}$ because $\overline {5}$ cannot trigger the reactions. So, given ***f***_*a*_, CRN 1 is not self-sustaining with respect to ***ξ***_*c*_.

Finally, in many cases, it is worthwhile to distinguish reducible and irreducible self-sustainability:

- A self-sustaining CRN (given inflow ***f***, with respect to initial condition ***ξ***) is *reducible self-sustaining* if, by excluding some of the reactions in this CRN, there is no effect on its dynamics; otherwise, it is *irreducible self-sustaining*.

Therefore, given the inflow ***f***_*a*_, CRN 1 is actually irreducible self-sustaining with respect to the initial condition ***ξ***_*a*_, because if any reaction is excluded, its dynamics will change.

### Concepts related to self-sustainability

By specifying the inflow ***f*** and the initial condition ***ξ***, self-sustainability is well-defined. Nonetheless, we are often more interested in another related question: Does a CRN have the potential to be self-sustaining? But before we look at this question, we need to further clarify the definition of self-sustainability. We do this by explaining other related concepts, which are summarised in Table 1.

**Table 1:** Concepts related to self-sustainability

(i)	$\phantom {\dot {i}\!}{\boldsymbol {f'}}_{{\boldsymbol {\xi }},\boldsymbol {f}} = \boldsymbol {f}$	$\phantom {\dot {i}\!}{\boldsymbol {f'}}_{{\boldsymbol {\xi }},\boldsymbol {f}} \neq \boldsymbol {f}$	$\phantom {\dot {i}\!}{\boldsymbol {f'}}_{{\boldsymbol {\xi }},\boldsymbol {f}} = \boldsymbol {f}$	$\phantom {\dot {i}\!}{\boldsymbol {f'}}_{{\boldsymbol {\xi }},\boldsymbol {f}} \neq \boldsymbol {f}$	
(ii)	$\phantom {\dot {i}\!}{\boldsymbol {f'}}_{{\boldsymbol {\xi }_{0}},\boldsymbol {f}} = \boldsymbol {f}$	$\phantom {\dot {i}\!}{\boldsymbol {f'}}_{{\boldsymbol {\xi }_{0}},\boldsymbol {f}} = \boldsymbol {f}$	$\phantom {\dot {i}\!}{\boldsymbol {f'}}_{{\boldsymbol {\xi }_{0}},\boldsymbol {f}} \neq \boldsymbol {f}$	$\phantom {\dot {i}\!}{\boldsymbol {f'}}_{{\boldsymbol {\xi }_{0}},\boldsymbol {f}} \neq \boldsymbol {f}$	
				$\phantom {\dot {i}\!}\boldsymbol {f'}_{\boldsymbol {\xi },\boldsymbol {f}} = \boldsymbol {f'}_{\boldsymbol {\xi }_{0},\boldsymbol {f}}$	$\phantom {\dot {i}\!}\boldsymbol {f'}_{\boldsymbol {\xi },\boldsymbol {f}} \neq \boldsymbol {f'}_{\boldsymbol {\xi }_{0},\boldsymbol {f}}$
	trivial	self-sustaining	impossible	sequential	sequential + self-sustaining

Firstly, let us look at the first column labelled with “trivial”. There are two such trivial systems that we have just mentioned above: Given ***f***_*b*_, CRN 1 is trivial with respect to ***ξ***_*a*_; Given ***f***_*a*_, CRN 1 is trivial with respect to ***ξ***_*c*_. We call it trivial because the condition $\phantom {\dot {i}\!}{\boldsymbol {f'}}_{{\boldsymbol {\xi }},\boldsymbol {f}} = \boldsymbol {f}$ and $\phantom {\dot {i}\!}{\boldsymbol {f'}}_{{\boldsymbol {\xi }_{0}},\boldsymbol {f}} = \boldsymbol {f}$ together guarantee that no reaction occurs after the transient period.

**Fig. 2:**
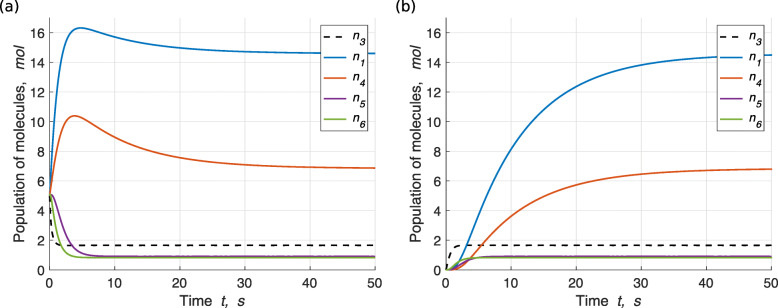
Mean-field dynamics of CRN 3 in CSTR. Note that the solvent molecule *n*_0_ is not shown. The reaction rate constants are *ω*_1_=0.6,*ω*_2_=1 and *ω*_3_=0.8. **a** The initial condition is ***ξ***_*d*_=(*n*_0_,*n*_1_,*n*_3_,*n*_4_,*n*_5_,*n*_6_)=(75,5,5,5,5,5). **b** The initial condition is ***ξ***_0_=(100,0,0,0,0,0). In both (**a**) and (**b**), the inflow is ***f***_*d*_=(*f*_0_,*f*_1_,*f*_3_,*f*_4_,*f*_5_,*f*_6_)=(8,0,2,0,0,0), and after the transient period, the outflow is $\boldsymbol {f'}_{\boldsymbol {\xi }_{d},\boldsymbol {f}_{d}} = \boldsymbol {f'}_{\boldsymbol {\xi }_{0},\boldsymbol {f}_{d}} \doteq (75.2, 14.6, 1.7, 6.8, 0.9, 0.8)$

Secondly, let us look at the fourth column labelled with “sequential”. Consider the following CRN 3 as an example,

3 $$  \left\{\begin{aligned} \overline{3} + \overline{3} & \rightarrow \overline{6} \\ \overline{6} & \rightarrow \overline{1} + \overline{5} \\ \overline{5} & \rightarrow \overline{1} + \overline{4} \end{aligned}\right. $$

with reaction rate constants predefined. Given the inflow ***f***_*d*_=(*f*_0_,*f*_1_,*f*_3_,*f*_4_,*f*_5_,*f*_6_)=(8,0,2,0,0,0), the mean-filed dynamics under the initial condition ***ξ***_*d*_=(*n*_0_,*n*_1_,*n*_3_,*n*_4_,*n*_5_,*n*_6_)=(75,5,5,5,5,5) is shown in Fig. 2a. We can see that $\phantom {\dot {i}\!}\boldsymbol {f'}_{\boldsymbol {\xi }_{d},\boldsymbol {f}_{d}} \neq \boldsymbol {f}_{d}$ is satisfied. On the other hand, under the zero initial condition ***ξ***_0_=(100,0,0,0,0,0), we have $\phantom {\dot {i}\!}\boldsymbol {f'}_{\boldsymbol {\xi }_{0},\boldsymbol {f}_{d}} \neq \boldsymbol {f}_{d}$ satisfied, as seen from Fig. 2b. Moreover, we have $\phantom {\dot {i}\!}\boldsymbol {f'}_{\boldsymbol {\xi }_{d},\boldsymbol {f}_{d}} = \boldsymbol {f'}_{\boldsymbol {\xi }_{0},\boldsymbol {f}_{d}}$, that is, no matter the initial condition is a non-zero condition ***ξ*** or a zero condition ***ξ***_0_, the outflows are the same. Therefore, CRN 3 is sequential with respect to the initial condition ***ξ***_*d*_, given the inflow ***f***_*d*_.

We call it sequential because, in this type of system, the products of some reactions constitute the reactants of other reactions in the next step, so on and so forth. Condition (i) and (ii) together guarantee that reactions in the tank continue to occur for all time and no molecule is needed initially to trigger the system (equivalently meaning that not all reactants can be regenerated by the system itself). The extra condition $\phantom {\dot {i}\!}\boldsymbol {f'}_{\boldsymbol {\xi },\boldsymbol {f}} = \boldsymbol {f'}_{\boldsymbol {\xi }_{0},\boldsymbol {f}}$ excludes the possibility that there are self-sustaining systems contained (that is also why in the fifth column, we have “sequential + self-sustaining”). Note that with the same logic as the irreducible self-sustainability, a sequential CRN can also be irreducible or reducible.

Finally, the case shown in the third column will not be possible, because if under the zero initial condition, some reactions continue to occur (as $\phantom {\dot {i}\!}\boldsymbol {f'}_{\boldsymbol {\xi }_{0},\boldsymbol {f}} \neq \boldsymbol {f}$ implies), it is not possible that under a non-zero condition ***ξ***, no reaction occurs (as $\phantom {\dot {i}\!}\boldsymbol {f'}_{\boldsymbol {\xi },\boldsymbol {f}} = \boldsymbol {f}$ implies).

## Does a CRN have the potential to be irreducible self-sustaining?

As mentioned, we might be more interested in another related question: Does a CRN have the potential to be self-sustaining? To show such potential, in principle we just need to find one particular inflow and one particular initial condition so that this CRN is self-sustaining. However, there is an infinite number of inflows and initial conditions that we could try, and we cannot guarantee that it does not have such potential unless we have tried all of the infinite conditions, which is clearly impossible. It thus deserves to study general properties of such systems so that we can screen them beforehand.

Also note that by combining an irreducible self-sustaining CRN with a trivial CRN, we can always obtain another self-sustaining CRN. It thus makes sense to only focus on irreducible self-sustaining CRNs. So in this section, we will discuss some of the general schemes (referring to Appendix A2 for more schemes) to discern whether a CRN has the potential to be irreducible self-sustaining.

### If a CRN has the potential to be irreducible self-sustaining, it must be “self-driven”

“Self-driven” is a property of CRN, meaning that for each reaction in this CRN, at least one type of its reactants comes from the products of other reactions in this CRN [35]. We prove (informally) this statement by considering a non-self-driven CRN. Take CRN 4 as an example,

4 $$  \left\{\begin{aligned} \overline{1} + \overline{2} & \rightarrow \overline{3} \\ \overline{1} + \overline{3} & \rightarrow \overline{4} \\ \overline{4} & \rightarrow \overline{2} + \overline{2} \\ \overline{5} + \overline{6} & \rightarrow \overline{11} \\ \overline{5} + \overline{11} & \rightarrow \overline{16} \\ \overline{16} & \rightarrow \overline{1} + \overline{15} \end{aligned}\right.  $$

where $\overline {5} + \overline {6} \rightarrow \overline {11}$ violates the condition of self-driven because neither of the reactants comes from the products of other reactions in CRN 4.

If the inflow does not contain both $\overline {5}$ and $\overline {6}$, the reaction $\overline {5} + \overline {6} \rightarrow \overline {11}$ cannot occur after the transient period no matter what the initial condition is. So, if we exclude this reaction, there will be no effect on the dynamics of this system. CRN 4 is thus reducible, no matter it is self-sustaining or not.

On the other hand, imagine that the inflow contains both $\overline {5}$ and $\overline {6}$. We can then equivalently consider that the inflows are $\overline {5}$ and $\overline {11}$, and exclude this reaction $\overline {5} + \overline {6} \rightarrow \overline {11}$. Similarly, we can further equivalently consider that the inflow is $\overline {16}$ and exclude the reaction $\overline {5} + \overline {11} \rightarrow \overline {16}$. This process can go on until the remaining CRN is self-driven. In this case, the first three reactions remain and the imagined inflows are $\overline {1}$ and $\overline {15}$. Now, given this inflow, if the remained CRN is not self-sustaining, the original CRN 4 cannot be self-sustaining either; On the other hand, if the remained CRN is self-sustaining, the original CRN 4 must be reducible self-sustaining. In either case, CRN 4 is not irreducible self-sustaining. The proof ends.

Note that not all of the self-driven CRNs have the potential to be irreducible self-sustaining, as shown in Appendix A3 where we have checked all self-driven CRNs up to 5 (i.e., the maximum molecule is $\overline {5}$) for whether each of them can be irreducible self-sustaining.

Therefore, to discern whether a CRN has the potential to be irreducible self-sustaining, we need to first divide it into self-driven and non-self-driven CRNs, and focus on the self-driven CRNs only.

### Hypothesis: two extra criteria guarantee a self-driven CRN to be irreducible self-sustaining

The hypothesis is that: If a self-driven CRN further satisfies

- the criterion for “overproduction”, i.e., there are some types of intermediate molecules, and the number of times it appears on the reactant side is less than that on the product side,
- and the criterion for “no-over-intake”, i.e., there is no type of intermediate molecules that the number of times it appears on the reactant side is larger than that on the product side,

then it is irreducible self-sustaining (where intermediate molecules refer to those appear on both the reactant side and the product side of the whole CRN). Those CRNs refer to the “self-replicating” CRNs defined in [35], meaning that at least one type of molecules in this CRN is produced more than consumed so that if the resource is unlimited, the number of molecules can grow exponentially.

To illustrate, take CRN 1 as an example. It satisfies the criteria for self-driven, overproduction and no-over-intake. And, as shown in Fig. 1a, it is irreducible self-sustaining with respect to initial condition ***ξ***_*a*_, given inflow ***f***_*a*_.

Plenty of CRNs that satisfy the three criteria have been checked, and all of them can be irreducible self-sustaining, given proper ***f*** and ***ξ***. But unfortunately, the attempt to prove this hypothesis failed.

Note that there are also lots of CRNs that do not satisfy the three criteria but is still able to self-sustain, referring to Appendix A2 for more examples.

## Irreducible self-sustaining systems in real chemistry and biology

In this section, we will list various chemical and biological systems that have the potential to be irreducible self-sustaining (all of them have been checked based on our definition). First of all, to embrace more realistic reactions, we further allow replacement reactions in our artificial chemistry framework, e.g., $\overline {3} + \overline {4} \rightarrow \overline {1} + \overline {6}$. It will not change the properties of the framework, though.

We begin with natural systems. The first example comes from the combustion of H_2_, a few important reaction steps in the early stages of the combustion [36, 37]:

$$\left\{\begin{array}{l} \overline{1} + \overline{6} \rightarrow \overline{3} + \overline{4} \\ \overline{2} + \overline{3} \rightarrow \overline{1} + \overline{4} \\ \overline{2} + \overline{4} \rightarrow \overline{1} + \overline{5} \end{array}\right.\quad \left|\begin{array}{ll} \overline{1} :~ \mathrm{H} &\quad\quad \overline{4} :~ \text{OH} \\ \overline{2} :~ \mathrm{H_{2}} &\quad\quad \overline{5} :~ \mathrm{H_{2}O} \\ \overline{3} :~ \mathrm{O}&\quad\quad \overline{6} :~ \mathrm{O_2} \end{array}\right. $$

Note that this CRN satisfies the criteria for self-driven, overproduction and no-over-intake. We see that if there is no $\overline {1}$ (H), $\overline {3}$ (O) or $\overline {4}$ (OH) present, no reaction can proceed. That is also why in reality $\overline {2}$ (H_2_) and $\overline {6}$ (O_2_) can be mixed without reacting. But if a small amount of $\overline {1}, \overline {3}$ or $\overline {4}$ is produced by a spark, for example, this CRN will be triggered and react very rapidly, in a self-replicating manner.

Another example is that atomic oxygen O combines into O_2_ in the upper atmosphere [36],

$$\left\{\begin{array}{l} \overline{1} + \overline{2} \rightarrow \overline{3}\\ \overline{1} + \overline{3} \rightarrow \overline{2} + \overline{2} \end{array}\right.\quad \left|\begin{array}{l} \overline{1} :~ \mathrm{O} \\ \overline{2} :~ \mathrm{O_{2}} \\ \overline{3} :~ \mathrm{O_{3}} \end{array}\right. $$

which also satisfies the criteria for self-driven, overproduction and no-over-intake.

Besides, a great number of metabolic pathways are able to self-sustain, e.g., the glyoxylate cycle [36],

$$\left\{\begin{array}{rl} \overline{12} + \overline{5} &\rightarrow \overline{4} + \overline{13} \\ \overline{11} + \overline{4} &\rightarrow \overline{8} + \overline{7} \\ \overline{8} &\rightarrow \overline{7} + \overline{1}\\ \overline{11} + \overline{1}& \rightarrow \overline{5} + \overline{7} \\ \overline{6} + \overline{7} &\rightarrow \overline{3} + \overline{10} \\ \overline{2} + \overline{3} &\rightarrow \overline{5} \end{array}\right.\!\!\!\quad \left|\begin{array}{ll} \overline{1} :~ \text{glyoxylate} &\text{~} \overline{7} :~ \text{CoA} \\ \overline{2} :~ \mathrm{H_{2}O} &\text{~} \overline{8} :~ \text{citrate} \\ \overline{3} :~ \text{fumarate} &\overline{10} :~ \mathrm{E.FADH_{2}} \\ \overline{4} :~ \text{oxaloacetate} &\overline{11}:~ \text{acetyl-CoA} \\ \overline{5} :~ \text{malate} &\overline{12} :~ \mathrm{NAD^{+}} \\ \overline{6} :~ \mathrm{E.FAD}&\overline{13} :~ \text{NADH} \end{array}\right. $$

which is an anabolic pathway occurring in various species; and the reverse citric acid cycle [11, 38],

$$\left\{\begin{array}{rl} \overline{4} + \overline{10} &\rightarrow \overline{14} \\ \overline{1} + \overline{14} &\rightarrow \overline{10} + \overline{5} \\ \overline{1} + \overline{5} &\rightarrow \overline{6}\\ \overline{6} + \overline{10} &\rightarrow \overline{12} + \overline{4} \\ \overline{1} + \overline{12} &\rightarrow \overline{10} + \overline{3} \\ \overline{1} + \overline{3} &\rightarrow \overline{4} \end{array}\right.\quad \left|\begin{array}{l} \overline{1} :~ \mathrm{CO}_{2} \\ \overline{3} :~ \text{pyruvate} \\ \overline{4} :~ \text{succinate} \\ \overline{5} :~ \alpha\text{-ketoglutarate} \\ \overline{6} :~ \text{citrate} \\ \overline{10} :~ \text{CoA} \\ \overline{12} :~ \text{acetyl-CoA} \\ \overline{14} :~ \text{succinyl-CoA} \end{array}\right. $$

which is used by some bacteria to produce carbon compounds from carbon dioxide, and also a candidate for prebiotic pathways. These two CRNs also satisfy the criteria for self-driven, overproduction and no-over-intake.

There are also examples that do not satisfy the criteria for self-driven, overproduction and no-over-intake, but are still able to self-sustain, e.g., the Calvin cycle in photosynthesis [39]:

$$\left\{\begin{array}{l} \overline{5} + \overline{1} \rightarrow \overline{3} + \overline{3} \\ \overline{3} + \overline{3} \rightarrow \overline{6} \\ \overline{3} + \overline{6} \rightarrow \overline{5} + \overline{4} \\ \overline{4} + \overline{6} \rightarrow \overline{5} + \overline{5} \end{array}\right.\quad \left|\begin{array}{l} \overline{1} :~ \mathrm{CO}_{2} \\ \overline{3} :~ \text{Glyceraldehyde 3-phosphate} \\ \overline{4} :~ \text{Erythrose 4-phosphate} \\ \overline{5} :~ \text{Ribulose 1,5-bisphosphate} \\ \overline{6} :~ \text{Fructose 6-phosphate} \end{array}\right. $$

Note that in these biochemical CRNs above, we used a few simplifications: (1) some reaction steps are catalysed by enzymes, which we did not consider here; (2) we did not consider some transformations between different molecular structures. Nevertheless, those simplifications will not change the overall structure of the CRNs. These CRNs listed above are just a drop in the ocean. There are much more natural CRNs that have the potential to be self-sustaining, some of which are huge, e.g., the whole metabolic network of *E. coli* [40].

Besides those natural systems, there are also lots of artificial ones. One of the most famous systems is the Belousov-Zhabotinsky reaction, which is a nonlinear chemical oscillator and can generate wave patterns. Based on the Oregonator model [41], it can be written as:

$$\left\{\begin{array}{rl} \overline{4} + \overline{10} &\rightarrow \overline{6} + \overline{8} \\ \overline{4} + \overline{8} &\rightarrow \overline{6} + \overline{6} \\ \overline{8} + \overline{10} &\rightarrow \overline{9} + \overline{9}\\ \overline{9} &\rightarrow \overline{1} + \overline{8} \\ \overline{8} + \overline{8} &\rightarrow \overline{6} + \overline{10} \\ \overline{1} + \overline{3} &\rightarrow \overline{4} \end{array}\right.\quad \left|\begin{array}{l} \overline{1} :~ \mathrm{Ce\ (IV)} \\ \overline{3} :~ \mathrm{CH}_{2}(COOH)_{2} \\ \overline{4} :~ \mathrm{Br^{-}} \\ \overline{6} :~ \text{HOBr}\\ \overline{8} :~ \mathrm{HBrO}_{2} \\ \overline{9} :~ \mathrm{BrO}_{2} \\ \overline{10} :~ \mathrm{BrO_{3}^{-}} \end{array}\right. $$

Another example is the cross-replicating RNA enzyme, achieved by Lincoln et al. [4, 42], which can be represented as:

$$\left\{\begin{array}{rl} \overline{2} + \overline{7} &\rightarrow \overline{9} \\ \overline{6} + \overline{9} &\rightarrow \overline{15} \\ \overline{15} &\rightarrow \overline{7} + \overline{8} \\ \overline{3} + \overline{8} &\rightarrow \overline{11} \\ \overline{4} + \overline{11} &\rightarrow \overline{15} \end{array}\right.\quad \left|\begin{array}{ll} \overline{2} :~ \text{substrate A} &\quad \text{~} \overline{7} :~ \text{enzyme E'} \\ \overline{3} :~ \text{substrate A'} &\quad \text{~} \overline{8} :~ \text{enzyme E} \\ \overline{4} :~ \text{substrate B'} &\quad \text{~} \overline{9} :~ \text{complex E'A} \\ \overline{6} :~ \text{substrate B} &\quad \overline{11} :~ \text{complex EA'} \\ &\quad \overline{15} :~ \text{complex E'E} \end{array}\right. $$

Similar RNA systems can also be found in other papers, such as [43–45].

Besides, there are also designed large-scale molecular networks that are able to self-sustain. Some were achieved by ribozymes [46], while some were achieved by peptides [47].

## Discussion

### On the definition

We first compare the definition in the chemical organisation theory with ours. Take CRN 1 and the following CRN 5 as an example:

5 $$  \left\{\begin{aligned} \overline{3} & \rightarrow \overline{1} + \overline{2} \\ \overline{4} & \rightarrow \overline{1} + \overline{3} \\ \overline{2} + \overline{2} & \rightarrow \overline{4} \end{aligned}\right.  $$

The two systems behave distinctly: On one hand, for CRN 5, given an inflow $\overline {2}$, all reactions continue to occur for all time whatever the initial condition is; On the other hand, for CRN 1, even if there is an inflow of $\overline {1}$, molecule $\overline {2}$ (or $\overline {3}$ or $\overline {4}$) still needs to be present initially, otherwise no reaction can occur. According to the chemical organisation theory, for both CRNs, the set of molecules $\overline {2}, \overline {3}$ and $\overline {4}$ is self-sustaining (because they are consumed and also produced by the system itself). However, they should be considered different since they behave differently. Our definition manages to distinguish them: CRN 1 is defined to be self-sustaining (given the inflow of $\overline {1}$ and the initial condition of $\overline {2}$), while CRN 5 is defined to be sequential.

Whether a CRN is self-sustaining is well-defined only when both the inflow and the initial condition are specified. The motivation is that self-sustainability of a CRN depends not only on the network itself but also on the external environment. For example, a living system, as it should be considered to be self-sustaining, cannot self-sustain if it is not in an appropriate environment.

Nevertheless, whether a CRN has the potential to be self-sustaining is often a more important question to ask, because those types of CRNs are building blocks of extant life [4, 6, 29] and might be what life began with [11, 18, 35, 39]. We thus discussed the general schemes to discern whether a CRN has such potential, and listed various such CRNs that we have already known in the real world.

There are good reasons why we define self-sustainability in the context of CSTR. First of all, to keep a chemical system open, CSTR is one of the simplest ways, in the sense that the equations that describe the dynamics in CSTR are simple, and the experimental settings are simple and commonly used [32, 33, 48]. But why do we need to consider open systems [49], namely those can exchange materials and energy with the environment? Because based on the second law of thermodynamics, any closed system will eventually reach the thermodynamic equilibrium, a state which has the maximum entropy (thus trivial) [3, 50]. The second reason we use CSTR is that the “flow chemistry” to which the setting of CSTR belongs has rapidly developed and become a reliable tool for developing synthetic chemistry and biology [33, 48]. Lastly, this definition gives a simple way to test whether a chemical system is self-sustaining both theoretically and empirically.

Finally, it is worthwhile to briefly mention the relation between our definition of self-sustainability with another popular term “autocatalysis”. Sometimes, “autocatalysis” is used in a vague and general sense to refer to a property of a chemical system that reactants can be produced by the system itself. While at other times, it refers to the rigorous definition in RAF theory that every reaction is catalysed by some molecule produced by the system or present in the food set. As the latter requires each reaction to be a catalysed reaction, the two meanings are distinct. Our definition does not fall into the RAF theory side, but could rather be considered to claim a formal and rigorous area in the vague domain of “autocatalysis” (refer to “Author’s reply 2.1” in section “Reviewers’ comments” for more discussions).

### On heredity

If a CRN is self-sustaining, it has preliminary heredity. As we mentioned, the definition of self-sustainability guarantees that if there is no trigger molecule (or called, seed) present initially, no reaction can occur (referring to CRN 1 for example). The trigger—one of the many intermediate molecules—starts one reaction, and all other intermediates needed will be produced afterwards in a collective manner. So, any one of the triggers contains the complete information of building the whole system, although all of the triggers will be present as long as the system is established. The whole set of triggers could be considered as a “holistic, limited hereditary replicator” [51] (refer to “Author’s response 1.2” in section “Reviewers’ comments” for more discussions on this point). Self-sustainability and heredity are thus not completely independent of each other.

Different trigger molecules may lead a system to different paths, which is another evidence that trigger molecules contain preliminary heredity. Imagine a CRN that consists of the following two CRNs,

6 $$  \left\{\begin{aligned} \overline{3} + \overline{6} & \rightarrow \overline{9} \\ \overline{3} + \overline{9} & \rightarrow \overline{12} \\ \overline{12} & \rightarrow \overline{6} + \overline{6} \end{aligned}\right.  $$

7 $$ \left\{\begin{aligned} \overline{3} + \overline{4} & \rightarrow \overline{7} \\ \overline{3} + \overline{7} & \rightarrow \overline{10} \\ \overline{10} & \rightarrow \overline{2} + \overline{8} \\ \overline{8} & \rightarrow \overline{4} + \overline{4} \end{aligned}\right.  $$

given an inflow of molecule $\overline {3}$ and nothing else initially (note that both CRN 6 and 7 have the potential to self-sustain). It is evident that initially, no reaction occurs. If a molecule $\overline {6}$ then enters the system, CRN 6 will be triggered, but not CRN 7; while if a molecule $\overline {4}$ enters the system, CRN 7 will be triggered, but not CRN 6. Different triggers thus lead the system to different paths. This is a very special property for those types of CRNs.

The fact that preliminary heredity comes from molecular trigger receives much less attention than it deserves. The molecular trigger/seed was only briefly mentioned as a special initial condition [14, 17], and discussed in the effects of finite numbers and stochastic fluctuations in chemical systems [52–54].

### Life and fire

One of the touchstones of a “good” definition of life is whether it can distinguish “life” from “fire” and other “dissipative structures” [1, 55]. Both life and fire seem to be able to self-sustain. The common argument to distinguish them is that fire simply dissipates available free energy while life employs free energy to produce order. This argument is, however, far from satisfying, because life is also dissipative if the surrounding environment is considered altogether, while fire (e.g., fire whirl) can also generate order [1].

Based on our definition though, some types of fire are self-sustaining while some are not. For example, as mentioned in the last section, the early stage of the combustion of H_2_ is a self-sustaining system. Those systems need molecular triggers (H, O and OH in this case) to proceed, so preliminary heredity is contained in those molecular triggers.

On the other hand, the combustion of carbon, as an example, is not self-sustaining. This combustion would involve one or several following reactions: C + O_2_→CO_2_,2C + O_2_→2CO and 2CO+O_2_→2CO_2_. Firstly, given a constant inflow of both C and O_2_, these reactions can continue to occur no matter what the initial condition is. It is thus sequential under this condition. Secondly, given any inflows other than including both C and O_2_, no reaction can occur. It is then a trivial system. So, the combustion of carbon can never be a self-sustaining system. Another thing to notice is that, for this type of fire, as long as the temperature is high enough (given both C and O_2_), the combustibles would be lighted spontaneously. It only needs a “physical” trigger (namely the temperature), rather than a molecular trigger as the self-sustaining fire—as well as life—does. No information can be stored anywhere in these fire systems, and there is thus no heredity at all.

So, based on our definition, “fire” can be divided into self-sustaining and non-self-sustaining fire systems. In the sense that life is also self-sustaining, self-sustaining fire systems and life can be put into the same category. As for where to draw the line between life and self-sustaining fire systems, it would be another different story (even, there might not be a line between them, but a continuum). One thing deserves to notice though: For those self-sustaining fire systems, the trigger molecules are relatively easy to be produced in the system itself (e.g., in the case of H_2_ combustion, the trigger H can be produced from H_2_ relatively easily, by a spark for example); while it is not the case for living systems.

## Conclusions

Whether a system is self-sustaining depends not only on the intrinsic property of the system itself but also on the external environment. Thus, our definition of self-sustainability requires specifying the inflow and initial condition, which are the abstraction of the external environment. Particularly, we simplify the external environment as a CSTR, a common setting that keeps chemical systems open (i.e., be able to exchange materials and energy with the environment). This setting provides a simple way to empirically discern whether or not a system can self-sustain; and in the meanwhile, it does not make our definition less general because whether a system has the potential to be self-sustaining only depends on the intrinsic property of the system indeed.

One of the distinct properties of self-sustaining systems is that the system can only proceed if molecular triggers (or called, seeds) are present initially. Molecular triggers are able to establish the whole chemical reaction system de novo, indicating that they carry the preliminary heredity of the system.

Based on our definition, self-sustaining fire systems can be distinguished from other fire systems, where the former requires molecular triggers to proceed and the latter does not. Thus, we can at least distinguish life from non-self-sustaining fire systems, although distinguishing life from self-sustaining fire (or other dissipative) systems remains an open question.

Precisely defining self-sustainability is not only theoretically important but also provides useful insights into what types of systems we should look for in the studies of origins of life, building synthetic living systems, etc. The general properties and the various real-life examples of self-sustaining systems we have discussed not only indicate that these systems are not uncommon, but also provide practical guidance on how to construct and detect such systems in real biology and chemistry.

## Reviewers’ comments

**Reviewer’s report 1**

**Wentao Ma**, College of Life Sciences, Wuhan University, China.

**Reviewer comments 1.1:***This is an interesting effort to clarify the meaning of “self-sustaining”, which is no doubt significant for us to appreciate the concept of life, but the author should make more illustration on the relationship between the chemical systems he exemplified and real living systems.In this paper, Dr. Liu discussed, concerning the concept of life, an interesting issue: What is a self-sustaining chemical system? The analysis appears convincing and the conclusion is also interesting. However, I have some remarks on the paper. Since the topic is concerning the concept of life, first, a relevant clarification is necessary. As the author mentioned, NASA provided a relatively authoritative definition: “Life is a self-sustaining chemical capable of undergoing Darwinian evolution”. In a previous paper of my own (The essence of life, Biology Direct, 2016, 11:49), I noted that for the two aspects of life, self-sustaining and Darwinian evolution, the former is in respect of an individual (or entity), whereas the latter is in respect of a lineage (from the level of population to that of species and that above) - or rather, in respect of the form of the individual. Just image, how can an individual system undergoing Darwinian evolution? Therefore, I concluded, the definition of life should be split, as some expression like: “A life form is a matter form capable of undergoing Darwinian evolution; a living entity is a self-sustaining chemical system - in nature, it results from the Darwinian evolution and might engage into further Darwinian evolution”. But this splitting definition, as I also admitted, is somewhat complex to understand. For a compromise, here I would like to accept the definition: “Life is a sort of self-sustaining chemical systems capable of undergoing Darwinian evolution”. In this definition, by introducing the key phrase “a sort of”, we can appreciate “Darwinian evolution” is in regard of some kind of entities, whereas “self-sustaining remains” in regard of individual entities - i.e., just as we call it, living things. In fact, the new definition I propose here is to an extent just inspired by the analysis in the present paper. As the author concluded, there are many kinds of self-sustaining systems (“not uncommon”) - so life is just one sort of them. The point is: life is different from other self-sustaining systems by the other characteristic aspect - capable of undergoing Darwinian evolution. If so, I think this also offers an answer to the author’s question: How to distinguish life from self-sustaining fire (or other dissipative) systems?*

**Author’s response 1.1:** I am grateful for the reviewer for his comments, appreciation and suggestions. I will try to give my thoughts. The definition of life is, of course, an essential question, but way beyond this paper to answer. Instead, I intend to give a formal and rigorous definition of self-sustainability that is explicitly included in the working definition of life from NASA and that serves as one of the two essential properties of life (the other is Darwin evolution as also pointed out by the reviewer). However, in the literature, there is no rigorous and satisfying definition of self-sustainability (some related concepts are discussed in this paper). This is the very motivation of this work, so I only touched the other aspect of life (the Darwinian /genetics /information side) by linking self-sustainability with preliminary heredity. As pointed out by the reviewer, life would be a self-sustaining system with extra criteria. If self-sustainability is now rigorously defined, the next steps to define life would be easier.

I am really happy that this paper can give some inspirations to the reviewer. I totally agree that self- sustainability and Darwinian evolution should be considered separately; and self-sustainability implies individuality while evolution implies interactions at the level of population. But I think adding “sort of” into the definition of life is still not satisfying, which makes the definition hand-waving and vague. But this kind of discussions or the definition of life certainly deserves a whole paper if not many more.

**Reviewer comments 1.2:***About the capability of undergoing Darwinian evolution, we should appreciate the author’s effort to associate self-sustainability with heredity. As we know, heredity is one prerequisite of Darwinian evolution. The attempt to connect the trigger molecules to some preliminary heredity is attractive and somehow reasonable. But I think the author should make a more detailed annotation on the difference between this preliminary heredity (limited heredity) and real heredity in living things (unlimited heredity) - it is in fact just the unlimited heredity that makes Darwinian evolution possible, as highlighted by Szathmary and coworkers. Actually, in another paper of mine (What does “the RNA world” mean to “the origin of life”? Life, 2017,7:49), I stated that the ‘self-’ in the self-sustainment for life is just defined by its genetic information, carried on the genetic molecules (mainly DNA). Here, it seems that the information is just carried by the trigger molecules. That is, if the heredity mentioned by the author is comparable with the real heredity, it appears that the genetic molecules are just the trigger molecules in living things (but note that in the concrete scenario, some maternal mRNA are also necessary to trigger a new round ontogenesis). Similarly, when describing the self-sustainability in chemical systems (CRN), the author should make more efforts to connect real living things, especially considering that the author aimed for a clearer understanding on the concept of life. For example, for the situation in real living systems, the key components are enzymes (encoded by genetic information) instead of reactants—as mentioned by the author, for simplification, enzymes were neglected when exemplifying the metabolic cases (i.e., the glyoxylate cycle, the reverse citric acid cycle and the Calvin cycle), but I think that here is just a chance for the author to talk more about the situation in true living systems. As another example, in a living cell, genetic molecules (mainly DNA), the “trigger” of the self-sustaining system, are neither consumed nor produced (unless for cell division), which is apparently different from the CRN described by the author. So forth and so on. I think the author should comment on things like these.All in all, this is an interesting effort to clarify the meaning of “self-sustaining”, which is no doubt significant for us to appreciate the concept of life, but the author should make more illustration on the relationship between the chemical systems he exemplified and real living systems.*

**Author’s response 1.2:** Thanks very much for appreciating the linking between self-sustainability and heredity. Yes, I do think the preliminary heredity that is carried by the trigger molecules corresponds to the limited heredity as Szathmary highlighted. Also, I do think the genetic molecules are just the trigger molecules in living things.

Nevertheless, the transition from limited to unlimited heredity deserves a close look, regarding the reviewer’s concern. In my opinion, the transition deeply relates to the number of possible configurations of the trigger molecules (or a group of trigger molecules). For example, (1) oxygen gas O_2_ is the trigger molecule in the exemplified system (atomic oxygen O combines into O_2_ in the upper atmosphere), but O_2_ only has one configuration and the heredity it carries is thus limited. (2) For larger molecules (imagine a crystal), if served as the trigger, they could have different configurations such as different isomers, left-handed or right-handed, the heredity they carry would be more but still finite and limited. (3) For DNA, RNA-like molecules, their length can be increased, their based pairs can be switched or replaced, so they basically have an infinite number of configurations which makes the heredity unlimited. The trigger molecules in (1) and (2) are constrained by their intrinsic physics which forbids extra configurations, but for RNA-like molecules, the physical constraint is just relaxed.

Another point to notice is that, we often consider all the possible RNAs for a bacterial species (for example) altogether, because no matter what particular RNA molecule, it can always trigger the similar chemical reactions in the body of the bacteria. This is also why RNA carries unlimited information, because we are basically talking about an infinite number of particular molecules. For oxygen or crystals as above, we simply cannot do that, as there is no such different oxygen gas or crystal. So, in my opinion, the limited and unlimited heredity is in principle the same in the sense of the underlying chemical system, but it appears different due to the different physics inside the molecules.

These discussions are interesting and important, but I thought they were beyond the point of defining self-sustainability. Nevertheless, in order to also give the credits to the reviewer for raising this issue, I added a sentence in “On Heredity” section to guide the reader here for more details.

For the last issue raised by the reviewer that “genetic molecules (mainly DNA) are neither consumed nor produced (unless for cell division)”, I actually have different opinions: DNA does “consumed” and “regenerated”. In the process of DNA being translated into mRNA under the help of enzyme RNA polymerase, this DNA segment and RNA polymerase (and part of the newly-formed mRNA) are first transformed into a different molecule, and transformed back when the process is done. In this process, the DNA segment and RNA polymerase basically both serve as catalysts. It is just very easy to neglect the transformation of DNA, because only a tiny segment of DNA is transformed and it does not even change positions after transforming back. For cell division, it is more obvious that DNA is consumed and regenerated.All in all, thanks again for the reviewer’s detailed comments and efforts.

**Reviewer’s report 2**

**David Baum**, Department of Botany, University of Wisconsin-Madison, USA; Wisconsin Institute for Discovery, University of Wisconsin-Madison, USA.

**Reviewer comments 2.0:***This paper provides a formal treatment and definition of the property of self-sustenance, often articulated as a key property of life. Liu uses a mathematical abstraction of a chemical reaction network (CRN) in a CSTR and develops a 2-parted definition which basically states that a CRN is self-sustaining if reactions happen in the reactor (indicated by a steady state outflow that differs in composition from the inflow) and these reactions require seeding by chemicals that are not in the inflow (i.e., if the reactor is empty to start, the outflow converges to the inflow). The author provides some heuristics for evaluating whether a CRN has the potential to be self-sustaining and also lists a number of well-known networks that satisfy his criterion.There are several good aspects of this paper. I like the of idea of modeling CRNs in a CSTR and then identifying key signatures of self-sustenance, I agree that the need for a trigger chemical, what is more commonly called a “seed”, can be seen as a characteristic of self-sustaining systems, and I appreciate the point about self-sustenance entailing proto-heritability. That said, I see a number of major weaknesses in the paper. For example, the mathematical formalism is clunky and laborious to work through, the model invokes only unidirectional reactions when reversible reactions are the norm, and the framing in terms of existing “definitions” of self-sustenance seems contrived (neither Benner nor Luisi really “define” self-sustenance). The work also needs to compare/contrast self-sustenance with autocatalysis, the more common concept for evaluating the life-likeness of a CRN. I do not see clear value in the long list of example networks and the section of fire is pretty shallow.Overall, the core idea of diagnosing self-sustenance based on dynamic behavior in a CSTR is good, but the execution would be much improved.*

**Author’s response 2.0:** Thanks the reviewer very much for the appreciation of this work, and the concerns/issues raised here. I will try to answer them one by one.

Please see Author’s reply 2.6 for the issue about uni-direction.

I framed this paper around self-sustainability is exactly because: NASA’s working definition of life (Benner 2010) explicitly said “life is a self-sustaining chemical system capable of undergoing Darwinian evolution”, but without mentioning what is “self-sustaining”; Luisi’s “autopoiesis” explicitly included the term “self-sustaining” but did not mention what is “self-sustaining” neither. It is not a straightforward and self-evident term. This issue also relates to the relation with “autocatalysis”. Please see Author’s reply 2.1 for more.

As for why I listed lots of examples and mentioned fire: The point of this paper is to (1) introduce the definition and the framework, (2) make the readers be aware of those self-sustaining systems which are not uncommon (this is why I introduced examples in various areas such as inorganic chemistry, biochemistry and metabolism), and (3) explain how the trigger molecule can be related to heredity. The third point is why I had a section to discuss fire, which I think is intriguing. As fire can grow, “replicate” and dynamically maintain its structure, then why should not it be considered as life? The classic answer to this question is that fire is a dissipative system but life should not be. This work provides a different angle: firstly, fires should be distinguished into non-self-sustaining fires and self-sustaining fires (the latter is more closely related to life); secondly, the trigger molecule for self-sustaining fire systems is easy to be produced (as the example mentioned, atom H for H_2_ combustion) but this is not the case for life (e.g., DNA molecules).

**Reviewer comments 2.1:***The paper is framed around “self-sustenance”, but never clarifies how this relates to the much more common term (in chemistry if not astrobiology), “autocatalysis”. I believe that autocatalytic systems need not be self-sustaining (in your sense) because they can emerge without seeding. However, as far as I can tell, a self-sustaining CRN must be autocatalytic making self-sustenance a special case of autocatalysis that arises when one or more seed chemical is needed to trigger autocatalytic self-propagation. It would greatly strengthen the paper to explore this relation.*

**Author’s response 2.1:** Thanks for the comments. Autocatalysis indeed appears quite often in the literature, but unfortunately it is often used with ambiguity. Let us first look at RAF theory (that the reviewer mentioned a few times) where autocatalysis is defined to be the case where every reaction is catalysed by some molecule produced by the system or present in the food set (ref 8 section 1 line 5, ref 18 section 1.1.1, ref 23 section 2.1). Note that in RAF theory, every reaction must be a catalytic reaction (while in our definition, there is no such requirement), i.e., A →B must be catalysed by another molecule C. Thus, a system is RAF requires that the catalysts are regenerated rather than the reactants. All the mathematical analysis is based on this formalisation, namely a chemical system is made of three rather than two parts: a set of molecules, a set of reactions, and a set of catalysts (e.g., ref 23 section 2.1). However, this requirement is too strong. Although most extant metabolic reactions are catalysed by enzymes, CRNs are generally not. Especially when we talk about the origin of life or the early stage of living systems, enzymes or catalysts should not be considered to be the default (they should emerge in the later stage of life). This is also the most criticised part of RAF theory. So in this sense, our definition is very different from the autocatalysis in RAF theory.

Nevertheless, I am guessing the reviewer concerned more about our definition with “the more general autocatalysis”, namely without the requirement of catalysed reactions. Note that besides the RAF theory (and its headstream: Kauffman’s autocatalytic set theory), there is no rigorous definition of “autocatalysis in the general sense”. The word “autocatalysis” was often used based on the intuition that reactants can also be produced by the system itself. That means, people can either (1) use “autocatalysis” in the sense of RAF theory (rigorous but requiring catalysts) or (2) use the word as a vague, intuitive and general term. If meaning (1) or (2) can be specified beforehand, the ambiguity will be gone, but unfortunately, it is often mixed-up and very confusing.

The reason I framed this paper around “self-sustaining” rather than “autocatalysis” is that: (1) I think “autocatalysis” is clear in principle (as meaning 1 and 2), as long as people can specify which they mean beforehand (but unfortunately not); and (2) the word “self-sustaining” appears in many important places even in NASA’s definition of life but it is not well-defined. There is one theory called Chemical Organisation Theory indeed defined “self-sustaining” rigorously, but I discussed the disadvantages in the Background section.

Now let us get back to the reviewer’s next comments “I believe that autocatalytic systems need not be self-sustaining...” If here “autocatalysis” refers to RAF theory, our definition of self-sustainability is definitely not a special case of autocatalysis, because no catalysed reaction is required. If here “autocatalysis” is used in a general sense, we could take it as a special case if we wish, but that should be OK, because the point is to define it rigorously, i.e., “autocatalysis” is vague and general, but “self-sustaining” is precise.

I added a paragraph in “On the definition” section to briefly explain how our definition relates to “autocatalysis”. The more details are referred here.

**Reviewer comments 2.2:***After thinking about your model and “definition” can I suggest defining a self-sustaining set as a set of chemicals that, given a specified food influx, will constantly make more members of the set if and only if seeded by at least one member of the set. This makes much more sense to me. In particular, it would directly justify the trigger/seed criterion: a system cannot be seen to be SELF-sustaining if none of its parts are needed for it to emerge! This would imply two minor tweaks to your framing. 1: the formose self-sustaining CRN contains chemicals 2-4, but not chemical 1 (formaldehyde), which is the food needed for self-sustenance. 2: the zero initial condition should be redefined as the food-only initial condition - the is a reactor started with the designated food solution.*

**Author’s response 2.2:** This suggested definition is in practical equivalent to our definition (except “a set of chemicals”, as it should be a set of reactions in my opinion, but that is minor). “Constantly make more members” and “requires seeds” are the natural consequences of our two conditions in the definition. It is indeed a very useful way to interpret the original definition. And I also totally agree with “a system cannot be seen to be SELF-sustaining if none of its parts are needed for it to emerge”. However, it seems strange if we impose the “trigger/seed” in the definition without explaining what a seed is beforehand. In the original definition on the other hand, the initial condition naturally serves as the seed and well-defined. But thanks very much for this comment and suggestion. I added a few sentences after the definition based on this suggestion, to help the reader interpret the definition.

**Reviewer comments 2.3:***You talk about the state after a “transient” period. I think it would be better to say that the first criterion is about converging to a state (steady or fluctuating) that deviates from the influx but the second says that it will converge to a steady state that is the same as the influx.*

**Author’s response 2.3:** Thanks very much for this. “Converging” is a nice word indeed. But “after a transient period” helps me keep the two nice equations in the definition which will be easily referred to later. An extra sentence is added below the definition based on this suggestion.

**Reviewer comments 2.4:***The principles for determining if a network could be self-sustaining is a stronger part of the paper, which you might consider expanding. I wonder how many of these principles have already been discussed in the literature and how many mirror the formal analyses in RAF theory?*

**Author’s response 2.4:** Thanks for the comments, interests and suggestion. The principles to determine if a CRN is self-sustaining definitely deserves further investigation (which is in the plan). There are indeed more principles discussed in the appendix. As mentioned, RAF theory explicitly requires catalysis, and its formal analysis also explicitly requires a catalyst set (ref 23 section 2.1). So the criteria I put forward are distinct from theirs. Nevertheless, we could see some similarities if we really force it. For example, the “self-driven” criterion in this paper requires that for each reaction, there is at least one reactant comes from the products of other reactions in the system. In RAF theory, the CATALYST for each reaction should either be produced by other reactions in the system or provided by the food set. If we force to exchange the word “reactant” and “catalysts”, they are quite similar, but normally we should not do that.

**Reviewer comments 2.5:***(All minor issues below). You mention RAF theory and say: “self-sustaining was referred to that each molecule in a chemical network can be produced starting from the food source”. This sounds like a “constructively auto-catalytic and F-generated” set, which is a special case of a RAF set that does not require any seeding and would NOT, therefore, qualify as self-sustaining by your criterion.*

**Author’s response 2.5:** In RAF theory, “self-sustaining” is used in a very vague and general sense indeed (please see ref 8 page 1 just under Introduction); while only “reflexively-autocatalytic” (either constructively or not) is precisely defined. Yes, a RAF set that does not require any seeding would not qualify as self-sustaining by my definition.

**Reviewer comments 2.6:***You assume unidirectional reactions, but chemical reactions are generally reversible, even if the rate constants in the forward and reverse direction differ greatly. At the least, can you explain why you did this and how a deviation from this assumption would alter your conclusions?*

**Author’s response 2.6:** For all the artificial chemistry examples I gave, I did only list one direction. But this is just for simplicity and for illustration purposes. The definition of self-sustainability will not be affected at all if we consider reversible reactions. This is also one advantage we can get from this definition because the only information we need to discern empirically whether a system is self-sustaining or not is the inflow and outflow, and what really happens inside the system (whether unidirectional, or reversible, or catalysed) can be analysed afterwards.

For the artificial chemistry model, namely those reactions written as $\overline {1}+\overline {3} \rightarrow \overline {4}$, I borrowed from the previously published work (ref 35). In this model, every reaction is reversible indeed, but we only write down the spontaneous direction. When the forward and the backward reaction constants differ greatly, only the spontaneous direction matters. But in principle, in this model, there is no problem to include both directions: If solving it by ODE, just add the equations for the reverse direction; if solving it numerically by our program, both directions are automatically considered. I chose this model to illustrate the definition, just for the purpose of simplicity. We can choose any theoretical model or real chemistry, and the definition will work.

**Reviewer comments 2.7:***The model feels overly cluttered with complex extra notation and often presents equations without putting them into words. I’ll be honest, but it took me very many hours to understand your approach. For example, it will help to provide a verbal descriptions of the criteria: “for a given inflow and starting condition, the steady state outflow differs from the inflow while it would have been the same as the inflow in the case that the reactor was initiated with just solvent.”*

**Author’s response 2.7:** Thanks very much for this suggestion. I have added verbal explanations for some equations.

**Reviewer comments 2.8:***If N is a count of molecules and you use ODEs, aren’t you implying that you can have a fractional number of molecules? Not a big deal, but worth stating the assumption that there are many molecules.*

**Author’s response 2.8:** Yes, definitely we can have a fractional number of molecules as it is in the unit of *mol*. I choose *N* as the population rather than concentration is to keep the units of the reaction constant of the second-order reaction (synthesis reaction) and the first-order reaction (decomposition reaction) identical. It is easier to compare the effects of the reaction constants, although it does not matter for the definition.

**Reviewer comments 2.9:**You use the term “trigger” but I think “seed” is better. See for example Vasas et al. (2012) or Peng *et al* (2020, ArXiv)..

**Author’s response 2.9:** Thanks for this suggestion. I think “trigger” gives more “physical feelings” while “seed” gives some “vital feeling”. I do not want to impose any living-related feelings here, as self-sustainability does not imply life automatically. But I added “seed” here and there to make that link.

**Reviewer comments 2.10:***Is “sequential” the best term? I understand the motivation: one can see steady changes over time in a system because there are sequences of reactions. But really the point is that these CRNs do not allow one to identify a food such that the system will only emerge if seeded. So maybe you don’t need a term, just say they are not-self-sustaining.*

**Author’s response 2.10:** Thanks for the comment. I think it makes sense to distinguish “trivial” and “sequential” (refer to table 1), both of which should not be considered “self-sustaining” but have a bit different properties.

**Reviewer comments 2.11:***In the “Hypothesis” section. You use the term “self-replicating.” Is that an error?*

**Author’s response 2.11:** Thanks for pointing this out. It is not an error but a poor legacy from a previous paper. The term “self-replicating” was used in the previous paper (ref 35) to refer to a particular property of a CRN, i.e., at least one type of molecules in this CRN is produced more than consumed. I admit that it may not be a good term to use in that paper, but here it is just for a reference and used once.

## Supplementary information

**Additional file 1** Appendix. It is the appendix to the main text. Each section shows different technical details, which has been referred to in the corresponding position in the main text.

## Data Availability

All data generated or analysed during this study are included in this published article and its supplementary information files.

## Abbreviations

CRN: chemical reaction network
CSTR: continuous-flow stirred tank reactor
ODE: ordinary differential equation
RAF: reflexively autocatalytic and food-generated
