# Supplementary material for: On the definition of a self-sustaining chemical reaction system and its role in heredity
Source: Biol Direct. 2020 Oct 6;15:15. doi: 10.1186/s13062-020-00269-0 (PMC7541320; doi:10.1186/s13062-020-00269-0)
Supplement: Supplementary file 1 — Additional file 1 Appendix. It is the appendix to the main text. Each section shows different technical details, which has been referred to in the corresponding position in the main text. [file 13062_2020_269_MOESM1_ESM.pdf]

Appendix to:
On the definition of a self-sustaining chemical reaction system and its role in heredity

Yu Liu

Institut Mittag-Leffler, Auravägen 17, 182 60 Djursholm, Sweden

A1. Derivation of ODEs Eq. (2) in the main text

The whole chemical system is in CSTR, so there is a predefined constant inflow rate for each molecule species $\bar{i}$ (including the solvent molecule $\bar{0}$), f_i (mol/s).

Then, we make two assumptions: all molecules are uniformly distributed, and all chemical species have the same molar volume α (L/mol). Note that the overall outflowing solution is the well-stirred solution in the tank. So, by denoting the overall outflow rate as F' (mol/s), the outflow rate for each molecule species $\bar{i}$ is $F'n_i/N$ where n_i is the population (mol) of molecule $\bar{i}$ in the tank, and $N = \sum_i n_i$ is the total population. We will explain how to calculate F' soon.

Reaction rate r. We first explain how to calculate the reaction rate of the ith reaction of CRN (1) in the main text, r_i (mol/s). By transition state theory, we can consider a synthesis reaction $A + B \to C$ as

$$A + B \rightleftharpoons M^{\ddagger} \xrightarrow{k^{\ddagger}} C$$

where $M^{\ddagger}$ is an activated complex that is in a rapid pre-equilibrium, and $k^{\ddagger}$ (in the unit of $1/s$) is the reaction rate constant of the latter process. The equilibrium constant $K^{\ddagger}$ for this pre-equilibrium is (1)

$$K^{\ddagger} = \frac{a_{M^{\ddagger}}}{a_A \cdot a_B} = \frac{[M^{\ddagger}]/c^{\circ}}{[A]/c^{\circ} \cdot [B]/c^{\circ}} = \frac{[M^{\ddagger}]c^{\circ}}{[A][B]}$$

where a_X (dimensionless) is the thermodynamic activity of chemical species X, $[X]$ is the molar concentration (mol/L) of X, and $c^{\circ} = 1$ mol/L is the standard molar concentration. So the reaction rate of $A + B \to C$ (in unit of $mol/L/s$) is (1)

$$\begin{aligned} \gamma &= k^{\ddagger}[M^{\ddagger}] = k^{\ddagger} \cdot K^{\ddagger} \cdot [A][B]/c^{\circ} \\ &= \nu^{\ddagger} \cdot \frac{RT}{N_A h} \frac{1}{\nu^{\ddagger}} e^{-\frac{\Delta G^{\ddagger}}{RT}} \cdot [A][B] \frac{1}{c^{\circ}} \\ &= \omega [A][B] \frac{1}{c^{\circ}} \end{aligned}$$

where $\nu^{\ddagger}$ is the vibration frequency of the activated complex $M^{\ddagger}$, which can be cancelled out, R is the gas constant, T is temperature, N_A is Avogadro constant, h is Planck constant, $\Delta G^{\ddagger}$ is the Gibbs energy of the activated complex, and we denoted all the terms before $[A]$ as ω.

As mentioned, all chemical species have the same molar volume α. We further assume that $\alpha = 0.018$ L/mol which is the molar volume of water. Then we have

$$\gamma = \frac{\omega}{c^{\circ}} \frac{n_A}{V} \frac{n_B}{V} = \frac{\omega}{c^{\circ}} \frac{n_A}{\alpha N} \frac{n_B}{\alpha N}$$

where n_X is the population of X, V is the total volume of the solution, and N is the population of all chemicals in the solution.

However, we want the reaction rate for the whole volume, i.e., the reaction rate in the unit of mol/s, denoted as r. So we should multiply γ by V, and then we have

$$r = \gamma V = \omega n_A n_B/(c^{\circ}\alpha N) = \omega n_A n_B/(vN) \qquad [A1]$$

where $v = c^{\circ}\alpha = 0.018$ is a dimensionless constant. Equation (A1) is the reaction rate we need for synthesis reactions. Note that different reactions have different ω.

On the other hand, a decomposition reaction $D \to E + F$ can be considered as

$$D \rightleftharpoons Q^{\ddagger} \to E + F$$

By similar derivations, we have

$$r = \omega n_D \qquad [A2]$$

The ultimate reason why there is no term vN in Eq. (A2) is that the equilibrium constant for the pre-equilibrium is

$$K^{\ddagger} = \frac{a_{Q^{\ddagger}}}{a_D} = \frac{[Q^{\ddagger}]/c^{\circ}}{[D]/c^{\circ}} = \frac{a_{Q^{\ddagger}}}{a_D} = \frac{[Q^{\ddagger}]}{[D]}$$

Overall outflow rate F'. Then, we explain how to calculate the overall outflow rate F'. The outflow in CSTR is to keep the volume of the reacting solution constant (2), so we have,

$$0 = \frac{dV}{dt} = F_V + \frac{dV'}{dt} - F'_V \qquad [A3]$$

where F_V is the overall volumetric inflow rate (L/s), F'_V is the overall volumetric outflow rate, and dV'/dt is the volume changing rate caused by the reactions. The simplest case is that $dV'/dt = 0$, and then we can set $F'_V = F_V$ so that the total volume never changes. But this is not the case for CRN (1).

It is straightforward to see that

$$\begin{aligned} \frac{dV'}{dt} &= \frac{d}{dt} \sum_i n_i V_{m,i} \\ &= \sum_i V_{m,i} \cdot \frac{dn_i}{dt} + \sum_i n_i \cdot \frac{dV_{m,i}}{dt} \end{aligned} \qquad [A4]$$

where $V_{m,i}$ is the molar volume of molecule species $\bar{i}$. As already mentioned above, we have assumed that each molecule species has an identical molar volume α, so Eq. (A4) becomes

$$\frac{dV'}{dt} = \alpha \sum_i \frac{dn_i}{dt}$$

The author declares no conflict of interest.

Abbreviation: CRN: chemical reaction network. CSTR: continuous-flow stirred tank reactor. ODE: ordinary differential equation.

E-mail: yu.ernest.liu@hotmail.com

Consequently, Eq. (A3)—the requirement from CSTR—becomes

$$0 = \alpha \sum_i f_i + \alpha \sum_i \frac{dn_i}{dt} - F'\alpha$$
$$\Rightarrow F' = F + \sum_i \frac{dn_i}{dt}$$

where $F = \sum_i f_i$ denotes the overall inflow rate (mol/s). Then, we only need to figure out the second term above. As reactions proceed, the number of molecules in the system changes indeed: The first reaction in CRN (1) occurs once, the number of molecule decreases by 1; the second reaction occurs once, the number of molecules decreases by one; and the third reaction occurs once, the number of molecules increases by one. So, we have

$$\sum_i \frac{dn_i}{dt} = -r_1 - r_2 + r_3$$

Finally, we have

$$F' = F - r_1 - r_2 + r_3$$

A2. Does a CRN have the potential to be irreducible self-sustaining? (continued)

A collectively catalytic CRN can never be irreducible self-sustaining. Collectively catalytic is a property of CRN (3), meaning that this CRN can transform reactants into products, and the populations of those intermediate molecules do not change. For example, CRN (A5) is collectively catalytic:

$$\begin{cases} \overline{5} \to \overline{1} + \overline{4} \\ \overline{6} \to \overline{1} + \overline{5} \\ \overline{2} + \overline{4} \to \overline{6} \end{cases} \qquad [A5]$$

It is a model of the citric acid cycle in cellular respiration where molecule $\overline{1}$ stands for carbon dioxide, $\overline{2}$ for acetyl-CoA, $\overline{4}$ for oxaloacetic acid, $\overline{5}$ for α-Ketoglutaric acid, and $\overline{6}$ for citric acid, respectively (3).

Given inflow $\boldsymbol{f}_g = (f_0, f_1, f_2, f_4, f_5, f_6) = (8, 0, 2, 0, 0, 0)$, Fig. A1 shows the dynamics under initial condition $\boldsymbol{\xi}_g = (n_0, n_1, n_2, n_4, n_5, n_6) = (75, 5, 5, 5, 5, 5)$. We see that after the transient period, $\boldsymbol{f}'_{\boldsymbol{\xi}_g, \boldsymbol{f}_g} = \boldsymbol{f}_g$. On the other hand, it is evident from the reaction equations (A5) that $\boldsymbol{f}'_{\boldsymbol{\xi}_0, \boldsymbol{f}_g} = \boldsymbol{f}_g$. So, given $\boldsymbol{f}_g$, CRN (A5) is trivial with respect to $\boldsymbol{\xi}_g$.

It is worthwhile to notice that although all reactants $\overline{4}$, $\overline{5}$ and $\overline{6}$ (except the inflow) can be produced by the three reactions in CRN (A5), they cannot be produced more than consumed (this CRN is thus "collectively catalytic" rather than "self-replicating" which was discussed in details in (3)). So due to the dilution of CSTR, $\overline{4}$, $\overline{5}$ and $\overline{6}$ presented initially will be finally washed away completely, leaving the molecules flowing in and out without any reaction occurring. Therefore, it is trivial instead of self-sustaining.

From the example we see that, for a collectively catalytic CRN, the whole system can only proceed if the intermediate molecules are present. On the other hand, there is always an outflow which makes intermediate molecules less and less. Eventually, all intermediate molecules will be washed away, and no reaction can occur any more. These two facts make collectively catalytic CRNs impossible to be self-sustaining.

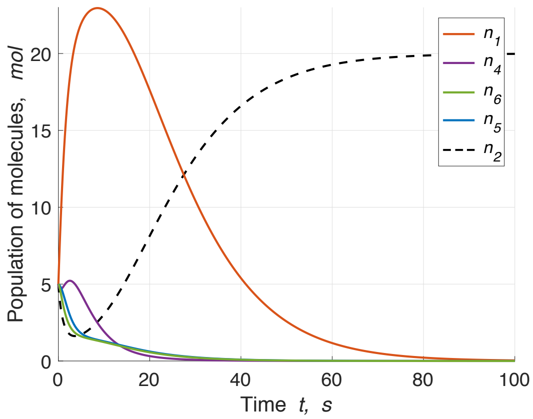

Fig. A1. Mean-field dynamics of CRN (A5) in CSTR. Note that the solvent molecule n_0 is not shown. The reaction rate constants are $\omega_1 = 0.9, \omega_2 = 1$ and $\omega_3 = 0.4$. The inflow is $\boldsymbol{f}_g = (f_0, f_1, f_2, f_4, f_5, f_6) = (8, 0, 2, 0, 0, 0)$. The initial condition is $\boldsymbol{\xi}_g = (n_0, n_1, n_2, n_4, n_5, n_6) = (75, 5, 5, 5, 5, 5)$. After the transient period, the outflow is $\boldsymbol{f}'_{\boldsymbol{\xi}_g, \boldsymbol{f}_g} = \boldsymbol{f}_g = (8, 0, 2, 0, 0, 0)$.

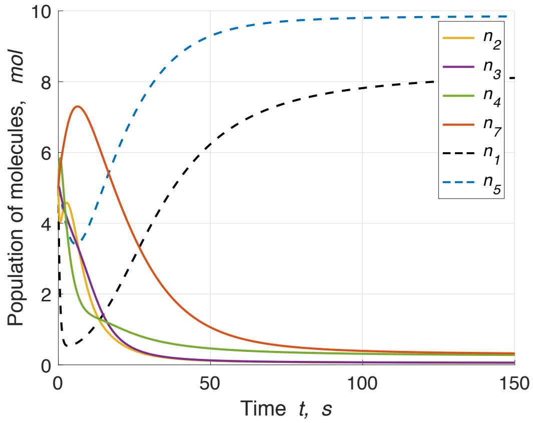

Fig. A2. Mean-field dynamics of CRN (A6) (formose reaction coupled with one side reaction) in CSTR. Note that the solvent molecule n_0 is not shown. The reaction rate constants are $\omega_1 = 0.4, \omega_2 = 0.35, \omega_3 = 0.25$ and $\omega_4 = 0.1$. The inflow is $\boldsymbol{f}_h = (f_0, f_1, f_2, f_3, f_4, f_5, f_7) = (8, 1, 0, 0, 0, 1, 0)$. The initial condition is $\boldsymbol{\xi}_h = (n_0, n_1, n_2, n_3, n_4, n_5, n_7) = (70, 5, 5, 5, 5, 5, 5)$. After the transient period, the outflow is $\boldsymbol{f}'_{\boldsymbol{\xi}_h, \boldsymbol{f}_h} \doteq (81.2, 8.3, 0.1, 0.1, 0.3, 9.9, 0.3)$.

Some self-driven CRNs have the potential to be irreducible self-sustaining even if they do not satisfy the criterion for overproduction and no-overintake. CRN (A6) is one of such examples:

$$\begin{cases} \overline{1} + \overline{2} \to \overline{3} \\ \overline{1} + \overline{3} \to \overline{4} \\ \overline{4} \to \overline{2} + \overline{2} \\ \overline{2} + \overline{5} \to \overline{7} \end{cases} \qquad [A6]$$

It is the formose reaction (CRN (1) in the main text) coupled with one side reaction. Intuitively, when the reaction rate constant of $\overline{2} + \overline{5} \to \overline{7}$, ω_4, is extremely small, its behaviour should be very similar with the original formose reaction which is self-replicating and self-sustaining as mentioned in the main text.

When ω_4 gets larger, self-replication gets slower (see details in the paper (4)). But as long as ω_4 is not larger than a critical

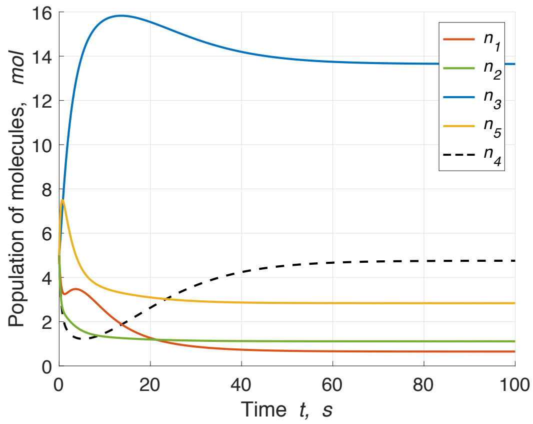

Fig. A3. Mean-field dynamics of CRN ([A7](#)). Note that the solvent molecule n_0 is not shown. The reaction rate constants are $\omega_1 = 0.8, \omega_2 = 1, \omega_3 = 0.3$, and $\omega_4 = 0.5$. The inflow is $\boldsymbol{f}_k = (f_0, f_1, f_2, f_3, f_4, f_5) = (8, 0, 0, 0, 2, 0)$. The initial condition is $\boldsymbol{\xi}_k = (n_0, n_1, n_2, n_3, n_4, n_5) = (75, 5, 5, 5, 5, 5)$. After the transient period, the outflow is $\boldsymbol{f}'_{\boldsymbol{\xi}_k, \boldsymbol{f}_k} \doteq (77.0, 0.6, 1.1, 13.6, 4.8, 2.8)$.

value, this CRN can still self-sustain, e.g., given inflow $\boldsymbol{f}_h$ and under initial condition $\boldsymbol{\xi}_h$, as shown in Fig. A2.

But note that if ω_4 is larger than a critical value, the system cannot self-sustain any more no matter what $\boldsymbol{f}$ and $\boldsymbol{\xi}$ are (4).

Self-sustaining CRNs do not need to have "resource molecules" explicitly. Resource molecules refer to those molecule types that only appear on the reactant side (3), e.g. molecule $\overline{2}$ in CRN (A5).

It is evident that in order to be self-sustaining, the resource molecule has to be included in the inflow. However, if a CRN has no resource molecule of this kind, it becomes less clear that which molecule has to be included in the inflow, or whether such CRNs can ever self-sustain. Here we show one example CRN (A7) that has no resource molecule of this kind, but is still able to self-sustain:

$$\begin{cases} \overline{2} \to \overline{1} + \overline{1} \\ \overline{1} + \overline{4} \to \overline{5} \\ \overline{2} + \overline{2} \to \overline{4} \\ \overline{5} \to \overline{2} + \overline{3} \end{cases} \qquad [A7]$$

Figure A3 shows its dynamics, given one inflow and one initial condition.

For any CRN, if the given inflow exceeds a certain threshold, this CRN can never self-sustain. It is straightforward to see that (1) if $\boldsymbol{f} = \boldsymbol{0}$, even though there are plenty of molecules initially, no reaction can occur after the transient period, so the CRN cannot self-sustain; and that (2) if $f_i > 0$ for every reactant, no matter what the initial condition is, all reactions can continue to occur and no trigger molecule is needed, so the CRN is sequential in this case. In order to self-sustain, $\boldsymbol{f}$ should thus be neither $\boldsymbol{0}$ nor that $f_i > 0$ for each reactant.

Furthermore, via plenty of simulations, it is observed that if the inflow is too large, anything interesting inside the system will be flowed away, and a trivial system will be achieved. I thus hypothesise that

- for any CRN, if $F > F_c$, a trivial system will always be achieved, where the threshold F_c is determined by the particular CRN, namely its topology and all of the reaction rate constants.

Table A1. U at different time points for various values of F.

F	U				
	t = 100	t = 500	t = 1,000	t = 5,000	t = 10,000
10	7.81	7.81	7.81	7.81	7.81
20	6.51	6.51	6.51	6.51	6.51
30	4.54	4.54	4.54	4.54	4.54
33	3.18	3.16	3.16	3.16	3.16
34	2.39	2.14	2.14	2.14	2.14
34.2	2.20	1.77	1.76	1.76	1.76
34.3	2.11	1.54	1.48	1.47	1.47
34.35	2.07	1.40	1.30	1.27	1.27
34.38	2.04	1.32	1.17	1.08	1.08
34.39	2.03	1.29	1.13	9.92E-1	9.90E-1*
34.4	2.02	1.26	1.08	8.67E-1	8.40E-1
34.5	1.93	9.72E-1	5.89E-1	1.06E-3	1.43E-7
35	1.47	1.20E-1	4.20E-3	0	0**
36	7.40E-1	9.70E-4	2.22E-7	0	0
37	3.30E-1	7.55E-6	0	0	0
40	2.27E-2	0	0	0	0
50	1.61E-6	0	0	0	0

* For simplicity, we use E-notation for scientific notation, e.g., 9.90×10^{-1} is denoted as 9.90E-1.
** If $U <$ 1E-7, then we set $U = 0$. Such small values are within the margin of error of numerical solutions.

Now we motivate this hypothesis. When the inflow is larger, the outflow is also larger. There will be a threshold beyond which the outflow of some intermediate molecules is larger than the rate they are produced by the reactions. So beyond the threshold, these intermediate molecules will be washed out eventually, and without them, the system cannot proceed any more. A trivial system is finally achieved. The fact that the threshold F_c is located where the outflow rate is larger than the production rate by the reactions explains why F_c should depend on the topology of the CRN and its reaction rate constants.

In the following, we took CRN (1) in the main text as an example to show the existence of F_c and how we can calculate it numerically. We will keep the proportion of each inflow the same, but change the overall inflow rate, i.e., keep f_i/F the same but change F.

Referring to Fig. 1(a) in the main text, I rewrite the inflow as $\boldsymbol{f} = (0.8, 0.2, 0, 0, 0) \cdot F$ where $F = 10$ in Fig. 1(a). By definition, a trivial system means that except for the inflowing molecules, all of the other molecules are washed away. For CRN (1), it means that $n_2 + n_3 + n_4 = 0$. Therefore, whether $n_2 + n_3 + n_4$ (denoted as U) eventually approaches zero indicates whether the system is trivial or not. So, I will check U at different time points for various F, varying from 10 to 50. The result is shown in Table A1.

To make it clearer, Fig. A4 shows the curves of U vs. F for the five different time points. The larger t is, the sharper the transition is. Evidently, when $t \to \infty$, the transition is abrupt, approximately at $F = 34.38$. Therefore, for CRN (1), in this particular case, the threshold F_c is approximately 34.38.

In general, F_c is determined by the topology of the CRN, the reaction rate constants, and the inflow compositions. But due to the strong nonlinear behaviours of the system, it is difficult to derive any analytical expression for F_c.

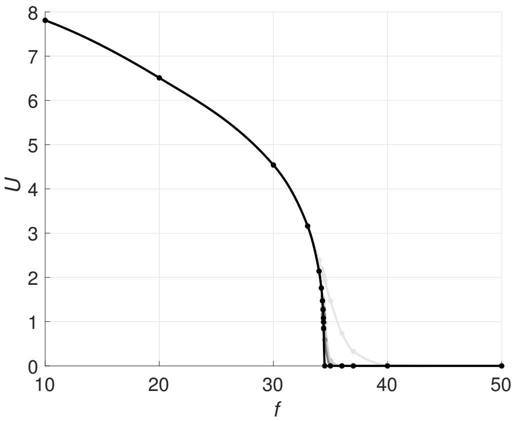

Fig. A4. Curves of U vs. F for the five different time points as shown in Table A1. Dots are the data points listed in Table A1, while solid lines are fitted curves. The darker the curve is, the larger t it corresponds to.

A3. Not all of the self-driven CRNs have the potential to be irreducible self-sustaining

Every possible CRN in which the maximum molecule is L has been checked, to see whether it has the potential to be irreducible self-sustaining. I just vary L from 1 up to 5, to show examples.

If $L = 1$, i.e. only $\overline{1}$ is the valid molecule, there will be no valid reaction (because $\overline{1} + \overline{1}$ even produces $\overline{2}$).

If $L = 2$, there will be two valid molecules $\overline{1}$ and $\overline{2}$, and two valid reactions $\overline{1} + \overline{1} \to \overline{2}$ and $\overline{2} \to \overline{1} + \overline{1}$ (we call one reaction and its reverse reaction as a *reaction pair*). So in total, we have

$$\binom{1}{0} \cdot 2^0 + \binom{1}{1} \cdot 2^1 = 1 + 2 = 3$$

possible CRNs. The first term corresponds to choosing zero reaction pair to be low-barrier (3) (i.e., with low Gibbs energy of activation). The second term corresponds to choosing one reaction pair to be low-barrier and either reaction to be spontaneous (i.e., the the sum of the Gibbs energy of formation of all reactants is greater than that of all products): So, one CRN is $\overline{1}+\overline{1} \to \overline{2}$, while the other is $\overline{2} \to \overline{1}+\overline{1}$. After checking, none of the three can self-sustain.

If $L = 3$, there will be three valid molecules $\overline{1}$, $\overline{2}$ and $\overline{3}$, and two reaction pairs $\overline{1} + \overline{1} \to \overline{2}$ and $\overline{1} + \overline{2} \to \overline{3}$ (for simplicity, we just write down the synthesis reaction to represent a reaction pair). So in total there are

$$\binom{2}{0} \cdot 2^0 + \binom{2}{1} \cdot 2^1 + \binom{2}{2} \cdot 2^2 = 1 + 4 + 4 = 9$$

possible CRNs. We list all of the nine in Table A2, where each curly bracket represents one CRN. After checking, none of them can self-sustain.

If $L = 4$, there will be four reaction pairs

$$\begin{aligned} \overline{1} + \overline{1} &\to \overline{2} \\ \overline{1} + \overline{2} &\to \overline{3} \\ \overline{1} + \overline{3} &\to \overline{4} \\ \overline{2} + \overline{2} &\to \overline{4} \end{aligned}$$

Table A2. All possible CRNs when $L = 3$.

$\binom{2}{0} \cdot 2^0$	$\binom{2}{1} \cdot 2^1$	$\binom{2}{2} \cdot 2^2$
$\varnothing$	$\{\overline{1}+\overline{1} \to \overline{2}$<br>$\{\ \overline{2} \to \overline{1}+\overline{1}$<br>$\{\overline{1}+\overline{2} \to \overline{3}$<br>$\{\ \overline{3} \to \overline{1}+\overline{2}$	$\begin{cases} \overline{1}+\overline{1} \to \overline{2} \\ \overline{1}+\overline{2} \to \overline{3} \end{cases}$<br>$\begin{cases} \overline{2} \to \overline{1}+\overline{1} \\ \overline{1}+\overline{2} \to \overline{3} \end{cases}$<br>$\begin{cases} \overline{1}+\overline{1} \to \overline{2} \\ \overline{3} \to \overline{1}+\overline{2} \end{cases}$<br>$\begin{cases} \overline{2} \to \overline{1}+\overline{1} \\ \overline{3} \to \overline{1}+\overline{2} \end{cases}$

Table A3. All possible self-driven CRNs when $L = 4$.

Number of possible CRNs	$\binom{4}{3} \cdot 2^3$	$\binom{4}{4} \cdot 2^4$
Number of possible self-driven CRNs	4	2
	$\begin{cases} \overline{2} \to \overline{1}+\overline{1} \\ \overline{1}+\overline{3} \to \overline{4} \\ \overline{4} \to \overline{2}+\overline{2} \end{cases}$ [A8]<br>$\begin{cases} \overline{1}+\overline{1} \to \overline{2} \\ \overline{4} \to \overline{1}+\overline{3} \\ \overline{2}+\overline{2} \to \overline{4} \end{cases}$<br>$\begin{cases} \overline{3} \to \overline{1}+\overline{2} \\ \overline{4} \to \overline{1}+\overline{3} \\ \overline{2}+\overline{2} \to \overline{4} \end{cases}$<br>$\begin{cases} \overline{1}+\overline{2} \to \overline{3} \\ \overline{1}+\overline{3} \to \overline{4} \\ \overline{4} \to \overline{2}+\overline{2} \end{cases}$ [A9]	$\begin{cases} \overline{2} \to \overline{1}+\overline{1} \\ \overline{3} \to \overline{1}+\overline{2} \\ \overline{4} \to \overline{1}+\overline{3} \\ \overline{2}+\overline{2} \to \overline{4} \end{cases}$<br>$\begin{cases} \overline{1}+\overline{1} \to \overline{2} \\ \overline{1}+\overline{2} \to \overline{3} \\ \overline{4} \to \overline{1}+\overline{3} \\ \overline{2}+\overline{2} \to \overline{4} \end{cases}$

and thus

$$\binom{4}{0} \cdot 2^0 + \binom{4}{1} \cdot 2^1 + \binom{4}{2} \cdot 2^2 + \binom{4}{3} \cdot 2^3 + \binom{4}{4} \cdot 2^4$$
$$= 1 + 8 + 24 + 32 + 16 = 81$$

possible CRNs. As already discussed in the main text, only self-driven CRNs have the potential to be self-sustaining. So, we only list all the self-driven CRNs, as shown in Table A3. The first column corresponds to the CRNs consisting of three reactions, and the second column corresponds to the CRNs consisting of four reactions. We checked all of them, only CRN (A8) and (A9) have the potential to self-sustain.

Table A4. All possible self-driven CRNs when $L = 5$ and $l = 3$.

$\begin{cases} \overline{2} \to \overline{1}+\overline{1} \\ \overline{1}+\overline{4} \to \overline{5} \\ \overline{5} \to \overline{2}+\overline{3} \end{cases}$ [A10]	$\begin{cases} \overline{1}+\overline{1} \to \overline{2} \\ \overline{5} \to \overline{1}+\overline{4} \\ \overline{2}+\overline{3} \to \overline{5} \end{cases}$
$\begin{cases} \overline{3} \to \overline{1}+\overline{2} \\ \overline{1}+\overline{4} \to \overline{5} \\ \overline{5} \to \overline{2}+\overline{3} \end{cases}$	$\begin{cases} \overline{1}+\overline{2} \to \overline{3} \\ \overline{5} \to \overline{1}+\overline{4} \\ \overline{2}+\overline{3} \to \overline{5} \end{cases}$
$\begin{cases} \overline{4} \to \overline{1}+\overline{3} \\ \overline{5} \to \overline{1}+\overline{4} \\ \overline{2}+\overline{3} \to \overline{5} \end{cases}$	$\begin{cases} \overline{1}+\overline{3} \to \overline{4} \\ \overline{1}+\overline{4} \to \overline{5} \\ \overline{5} \to \overline{2}+\overline{3} \end{cases}$
$\begin{cases} \overline{5} \to \overline{1}+\overline{4} \\ \overline{4} \to \overline{2}+\overline{2} \\ \overline{2}+\overline{3} \to \overline{5} \end{cases}$ [A11]	$\begin{cases} \overline{1}+\overline{4} \to \overline{5} \\ \overline{2}+\overline{2} \to \overline{4} \\ \overline{5} \to \overline{2}+\overline{3} \end{cases}$

As for $L = 5$, there are 6 valid reaction pairs

$$\overline{1}+\overline{1} \to \overline{2}$$
$$\overline{1}+\overline{2} \to \overline{3}$$
$$\overline{1}+\overline{3} \to \overline{4}$$
$$\overline{1}+\overline{4} \to \overline{5}$$
$$\overline{2}+\overline{2} \to \overline{4}$$
$$\overline{2}+\overline{3} \to \overline{5}$$

and in total

$$\sum_{l=0}^{6} \binom{6}{l} \cdot 2^l = 729 \qquad [A12]$$

possible CRNs. In fact, it is straightforward to see that the minimal self-driven CRN must consist of three reactions. So only when $l = 3, 4, 5, 6$, we can find self-driven CRNs.

Here, we only list all of the self-driven CRNs that consist of three ($l = 3$) or four ($l = 4$) reactions, beyond which (namely $l = 5$ and 6) there are too many CRNs to check comprehensively.

When $l = 3$, there are 8 self-driven CRNs, as shown in Table A4. Only CRN (A10) and (A11) have the potential to self-sustain. When $l = 4$, there are 28 self-driven CRNs, as shown in Table A5. There are 9 CRNs that have the potential to self-sustain: CRN (A13) − (A21).

1. Atkins P, de Paula J (2014) *Atkins' Physical Chemistry.* (Oxford University Press), 10th edition, pp. 249, 895, 899.
2. Schmidt LD (1998) *The Engineering of Chemical Reactions.* (Oxford University Press, New York), pp. 86–103.
3. Liu Y, Sumpter DJT (2018) Mathematical modeling reveals spontaneous emergence of self-replication in chemical reaction systems. *Journal of Biological Chemistry* 293(49):18854–18863.
4. Liu Y, Hjerpe D, Lundh T (2020) Side reactions do not completely disrupt linear self-replicating chemical reaction systems. *Artificial Life* 26(3).

Table A5. All possible self-driven CRNs when $L = 5$ and $l = 4$.

$\begin{cases}\bar{2}\to\bar{1}+\bar{1}\\ \bar{3}\to\bar{1}+\bar{2}\\ \bar{1}+\bar{4}\to\bar{5}\\ \bar{5}\to\bar{2}+\bar{3}\end{cases}$ [A13]	$\begin{cases}\bar{2}\to\bar{1}+\bar{1}\\ \bar{1}+\bar{2}\to\bar{3}\\ \bar{1}+\bar{4}\to\bar{5}\\ \bar{5}\to\bar{2}+\bar{3}\end{cases}$	$\begin{cases}\bar{1}+\bar{1}\to\bar{2}\\ \bar{3}\to\bar{1}+\bar{2}\\ \bar{1}+\bar{4}\to\bar{5}\\ \bar{5}\to\bar{2}+\bar{3}\end{cases}$	$\begin{cases}\bar{1}+\bar{1}\to\bar{2}\\ \bar{1}+\bar{2}\to\bar{3}\\ \bar{5}\to\bar{1}+\bar{4}\\ \bar{2}+\bar{3}\to\bar{5}\end{cases}$
$\begin{cases}\bar{2}\to\bar{1}+\bar{1}\\ \bar{1}+\bar{3}\to\bar{4}\\ \bar{1}+\bar{4}\to\bar{5}\\ \bar{4}\to\bar{2}+\bar{2}\end{cases}$ [A14]	$\begin{cases}\bar{1}+\bar{1}\to\bar{2}\\ \bar{4}\to\bar{1}+\bar{3}\\ \bar{1}+\bar{4}\to\bar{5}\\ \bar{2}+\bar{2}\to\bar{4}\end{cases}$	$\begin{cases}\bar{1}+\bar{1}\to\bar{2}\\ \bar{1}+\bar{3}\to\bar{4}\\ \bar{5}\to\bar{1}+\bar{4}\\ \bar{2}+\bar{3}\to\bar{5}\end{cases}$	$\begin{cases}\bar{2}\to\bar{1}+\bar{1}\\ \bar{1}+\bar{3}\to\bar{4}\\ \bar{4}\to\bar{2}+\bar{2}\\ \bar{2}+\bar{3}\to\bar{5}\end{cases}$ [A15]
$\begin{cases}\bar{1}+\bar{1}\to\bar{2}\\ \bar{4}\to\bar{1}+\bar{3}\\ \bar{2}+\bar{2}\to\bar{4}\\ \bar{2}+\bar{3}\to\bar{5}\end{cases}$	$\begin{cases}\bar{2}\to\bar{1}+\bar{1}\\ \bar{5}\to\bar{1}+\bar{4}\\ \bar{4}\to\bar{2}+\bar{2}\\ \bar{2}+\bar{3}\to\bar{5}\end{cases}$	$\begin{cases}\bar{2}\to\bar{1}+\bar{1}\\ \bar{1}+\bar{4}\to\bar{5}\\ \bar{2}+\bar{2}\to\bar{4}\\ \bar{5}\to\bar{2}+\bar{3}\end{cases}$ [A16]	$\begin{cases}\bar{1}+\bar{1}\to\bar{2}\\ \bar{5}\to\bar{1}+\bar{4}\\ \bar{4}\to\bar{2}+\bar{2}\\ \bar{2}+\bar{3}\to\bar{5}\end{cases}$ [A17]
$\begin{cases}\bar{1}+\bar{1}\to\bar{2}\\ \bar{5}\to\bar{1}+\bar{4}\\ \bar{2}+\bar{2}\to\bar{4}\\ \bar{2}+\bar{3}\to\bar{5}\end{cases}$	$\begin{cases}\bar{3}\to\bar{1}+\bar{2}\\ \bar{4}\to\bar{1}+\bar{3}\\ \bar{1}+\bar{4}\to\bar{5}\\ \bar{2}+\bar{2}\to\bar{4}\end{cases}$	$\begin{cases}\bar{1}+\bar{2}\to\bar{3}\\ \bar{1}+\bar{3}\to\bar{4}\\ \bar{1}+\bar{4}\to\bar{5}\\ \bar{4}\to\bar{2}+\bar{2}\end{cases}$	$\begin{cases}\bar{3}\to\bar{1}+\bar{2}\\ \bar{4}\to\bar{1}+\bar{3}\\ \bar{5}\to\bar{1}+\bar{4}\\ \bar{2}+\bar{3}\to\bar{5}\end{cases}$
$\begin{cases}\bar{3}\to\bar{1}+\bar{2}\\ \bar{1}+\bar{3}\to\bar{4}\\ \bar{1}+\bar{4}\to\bar{5}\\ \bar{5}\to\bar{2}+\bar{3}\end{cases}$	$\begin{cases}\bar{1}+\bar{2}\to\bar{3}\\ \bar{4}\to\bar{1}+\bar{3}\\ \bar{5}\to\bar{1}+\bar{4}\\ \bar{2}+\bar{3}\to\bar{5}\end{cases}$ [A18]	$\begin{cases}\bar{1}+\bar{2}\to\bar{3}\\ \bar{1}+\bar{3}\to\bar{4}\\ \bar{5}\to\bar{1}+\bar{4}\\ \bar{2}+\bar{3}\to\bar{5}\end{cases}$	$\begin{cases}\bar{1}+\bar{2}\to\bar{3}\\ \bar{1}+\bar{3}\to\bar{4}\\ \bar{1}+\bar{4}\to\bar{5}\\ \bar{5}\to\bar{2}+\bar{3}\end{cases}$ [A19]
$\begin{cases}\bar{3}\to\bar{1}+\bar{2}\\ \bar{4}\to\bar{1}+\bar{3}\\ \bar{2}+\bar{2}\to\bar{4}\\ \bar{2}+\bar{3}\to\bar{5}\end{cases}$	$\begin{cases}\bar{1}+\bar{2}\to\bar{3}\\ \bar{1}+\bar{3}\to\bar{4}\\ \bar{4}\to\bar{2}+\bar{2}\\ \bar{2}+\bar{3}\to\bar{5}\end{cases}$	$\begin{cases}\bar{1}+\bar{2}\to\bar{3}\\ \bar{1}+\bar{4}\to\bar{5}\\ \bar{2}+\bar{2}\to\bar{4}\\ \bar{5}\to\bar{2}+\bar{3}\end{cases}$	$\begin{cases}\bar{4}\to\bar{1}+\bar{3}\\ \bar{5}\to\bar{1}+\bar{4}\\ \bar{4}\to\bar{2}+\bar{2}\\ \bar{2}+\bar{3}\to\bar{5}\end{cases}$
$\begin{cases}\bar{4}\to\bar{1}+\bar{3}\\ \bar{1}+\bar{4}\to\bar{5}\\ \bar{2}+\bar{2}\to\bar{4}\\ \bar{5}\to\bar{2}+\bar{3}\end{cases}$	$\begin{cases}\bar{1}+\bar{3}\to\bar{4}\\ \bar{5}\to\bar{1}+\bar{4}\\ \bar{4}\to\bar{2}+\bar{2}\\ \bar{2}+\bar{3}\to\bar{5}\end{cases}$ [A20]	$\begin{cases}\bar{1}+\bar{3}\to\bar{4}\\ \bar{1}+\bar{4}\to\bar{5}\\ \bar{4}\to\bar{2}+\bar{2}\\ \bar{5}\to\bar{2}+\bar{3}\end{cases}$	$\begin{cases}\bar{1}+\bar{3}\to\bar{4}\\ \bar{1}+\bar{4}\to\bar{5}\\ \bar{2}+\bar{2}\to\bar{4}\\ \bar{5}\to\bar{2}+\bar{3}\end{cases}$ [A21]
